# Theoretical estimates on the expected number of mutations needed to reconstruct clonal lineage trees

**DOI:** 10.1093/bioadv/vbag264

**Published:** 2026-09-07

**Authors:** Nishat Anjum Bristy, Russell Schwartz

**Affiliations:** Ray and Stephanie Lane Computational Biology Department, Carnegie Mellon University, Pittsburgh, PA 15213, United States; Ray and Stephanie Lane Computational Biology Department, Carnegie Mellon University, Pittsburgh, PA 15213, United States; Department of Biological Sciences, Carnegie Mellon University, Pittsburgh, PA 15213, United States

## Abstract

**Motivation:**

Phylogenetics faces a growing challenge from increasingly large and complicated data sets enabled by ever-improving sequencing technologies. The issue is particularly acute for somatic evolution studies, such as cancer cell lineages, where single-cell data sets may include tens of thousands of mutations in hundreds of thousands of genetically distinct cells. Simultaneously, the biological complexity of somatic evolution has led to complex phylogeny methods that struggle to scale to even modest data sizes.

**Results:**

We explore the theoretical and empirical basis for one strategy for managing these large data sets: subsampling mutations for the computationally challenging phylogeny problem followed by faster placement of mutations on a putatively known guide tree. We specifically focus on determining the number of mutations sufficient to recover the true phylogeny at some level of resolution with high probability. We theoretically analyze variants of several common models that underlie popular tools for building clonal lineage trees. We further test these bounds through simulations of these models, extensions of them, and real biological datasets. The results suggest that modest numbers of mutations suffice to reconstruct clonal tree topologies for typical numbers of clones, supporting subsampling as a general strategy for managing the challenges of ever-growing data.

**Availability and implementation:**

All analysis code and scripts used for data simulation are implemented in Python 3 and available at https://github.com/CMUSchwartzLab/mutation-subsampling.git.

## 1 Introduction

Reconstructing evolutionary trees, i.e. phylogenetics, is a fundamental problem in biology aimed at understanding how different entities such as species, genes, or cells diversify from a common ancestor. Applications of phylogenetic trees have branched well beyond their classic use in species/organismal evolution (Nei and Kumar 2000, Semple *et al*. 2003) to cover applications in single-cell somatic evolution (Pennington *et al*. 2007) and cell lineage tracing (Woodworth *et al*. 2017). Phylogenetics has proven a particularly powerful tool for studies of somatic evolution in cancer progression (Schwartz and Schäffer 2017, Somarelli *et al*. 2017) and the theory and tools developed for that purpose have since helped make possible much broader study of somatic evolution in other diseases (Kennedy *et al*. 2012) and putatively healthy tissues (De 2011).

A growing problem in phylogenetic studies, at both species and somatic scales, is scaling to ever-larger data sets. At the species level, the field now needs to tackle databases of tens of thousands to even millions of organisms to be reconciled into single trees (Zaharias and Warnow 2022). For somatic evolution, single-cell datasets can reach into tens of thousands of single cells per sample (Svensson *et al*. 2018). Cancer genomes are particularly problematic because they frequently exhibit phenotypes of hypermutability that can lead to thousands of mutations per tumor distributed across a heterogeneous population of cells (Roberts and Gordenin 2014). While these vast data sets are crucial for shedding new light on somatic mutation processes, they are also algorithmically challenging for phylogenetic inference methods. At the same time, somatic evolution applications have the advantage over typical uses in species trees of a somewhat imprecise definition of taxa (typically species for organismal evolution but genetically similar subpopulations of cells called clones in the somatic case). Phylogenetics in cancer typically involves resolving a tree into the order of ten clones regardless of the number of cells, and one can opt to solve the problem at a lower resolution (fewer defined clones) if the data do not support finer resolution.

Many methods operate either directly on the reads or on a binary mutation matrix (entities × mutations), where each entry indicates presence or absence of a mutation, which are interpreted in terms of a simplified model of the evolutionary process. Many methods use a “perfect phylogeny” model, which assumes that a mutation is gained once and never lost, equivalent in practice to the “infinite sites” model that there are an infinite number of potentially mutable sites and so each can be mutated only once along the evolutionary history (Gusfield 1991). Under the perfect phylogeny and infinite-sites assumption, the exact phylogeny can be reconstructed in linear time. More complex models based on finite sites assumptions allow a site to be mutated multiple times along the evolutionary history. Models such as Dollo (Farris 1977) or *k*-Dollo (El-Kebir 2018) offer a compromise in which we assume a mutation is gained only once and allowed to be lost at most *k* times along the branches of the phylogeny. Full finite site models may allow arbitrary recurrent mutation or reversion.

While somatic phylogenetics drew inspiration and early methods from species phylogenetics, the biology and data characteristics are actually quite different and have led to widely divergent algorithms and inference challenges (Schwartz and Schäffer 2017). While the algorithmic work in species evolution has focused to a large degree on the challenge of scaling to larger numbers of taxa, the number of meaningfully different taxa (clones) is generally small in somatic clonal trees, on the order of ten, and the numbers of variant sites becomes the limiting factor in algorithmic tractability. Conversely, capturing somatic mutability accurately has required more complex variant models allowing for complex nested structural variations (SVs) (Eaton *et al*. 2018), copy number alterations (CNAs) (Chowdhury *et al*. 2014, Schwarz *et al*. 2014), and/or combinations of these with more classic single nucleotide variant (SNV) models (Fu *et al*. 2022, Sollier *et al*. 2023, Bristy *et al*. 2025). Algorithms for the most sophisticated variant models have worst-case exponential scaling with the number of variant sites, making scaling to large numbers of variants the more important challenge algorithmically in the somatic space. Together with tremendous advances in our abilty to acquire sequence data, this creates a scenario quite different from what is typically assumed in species phylogenetics: where we typically have an abundance of data but algorithms for sophisticated models scale too poorly to handle it.

The somatic evolution field has generally dealt with the problem of excessively large variant data sets heuristically. The most widely used approach is clustering mutations to a small number of equivalence classes that can then be treated as single weighted markers (Deshwar *et al*. 2015). While valuable, this approach depends on our ability to reliably separate co-occuring mutations by variant allele frequency (VAF) in bulk data or co-occurrance patterns in single-cell data, which can become increasingly difficult as noise and mutation burden grow. Another possibility is the use of consensus tree methods (Govek *et al*. 2018), allowing one to apply phylogeny methods that scale poorly in numbers of markers to subsets of the data and then synthesize the resulting family of trees into a consensus model. PhyDOSE (Weber *et al*. 2020) tackles the complementary optimization problem of choosing the number of cells to sequence in order to reconstruct reliable phylogenies. In this paper, we focus on a fourth strategy with precedent in species evolution (Erdos *et al*. 1999) that we have deployed in our prior work (Fu *et al*. 2022): using a subset of mutations to solve the hard problem of finding the correct clonal tree topology and then using faster algorithms for the easier problem of placing the remaining mutations onto that putatively known clonal tree. This can be thought of as a variation on the species tree strategy of using a subset of the data to form a reliable backbone tree followed by faster phylogenetic placement methods to place the remainder of the data on the backbone. The mutation sampling version of this problem hinges on the conjecture that if we have a mutation data set that is large compared to the number of clones we need to resolve, then we should be able to find the right tree with a small sample of the data. Erdos *et al*. (1999) showed under a standard simplified phylogeny model (Neyman 1971) that one can bound the number of mutations needed to identify all edges in a tree with high probability. This paper seeks to put that conjecture on a firmer theoretical basis for the somatic evolution case by extending the Erdos et al. work to models better approximating the biology of clone trees and to explore the practical question of how many mutations we need to use in our phylogeny inference to get the right tree topology at a given clonal resolution.

In the remainder of the paper, we assess this conjecture through theoretical and empirical methods. We first determine the bounds for the number of mutations sufficient and necessary for reconstructing evolutionary histories for several variations of phylogeny inference from point mutations (SNVs). For each, we derive theoretical bounds to establish how many mutations one needs to derive the correct tree with confidence. We use experiments on simulated data for these models and more complex variants of them to show that these theoretical bounds yield good results in practice. Our results support the use of the subsampling strategy as a basis for fast and accurate somatic evolution inference in the face of large data sets and costly phylogeny models and provide a basis for further study into extending this strategy of mutation subsampling and placement to more complicated evolutionary and mutation models.

## 2 Preliminaries

We first define some general terminology we will use throughout this paper; other terminology will be introduced as needed. Throughout the paper, we use these terms in related but context-dependent senses: nodes and leaves refer to the graph-theoretic structure of a tree, taxa refers primarily to the observed data in species phylogenetics, and clones refers to the cellular subpopulations relevant in somatic or tumor phylogenies, where the observed data may correspond to cells or inferred clonal sub-populations.

All considered phylogenetic trees T are binary trees with node set V, edge set E, and leaf set *L*. Let m=|E| denote the number of edges and n=|L| the number of leaves. We represent mutation data by a binary matrix $M\in {\left\{0,1\right\}}^{n\times s}$, whose rows correspond to entities (e.g. species, observed cells, or clones) and columns correspond to mutations, where $M_{i,j}=1$ indicates that mutation *j* is present in entity *i*. Throughout this section, we treat *M* as generated from an underlying phylogenetic tree *T* according to one of the evolutionary models considered (perfect, 1-Dollo, or coalescent). Let $\ell_{1},\ldots ,\ell_{m}>0$ be the edge lengths of T, and let $L_{\text{tot}}=\sum_{i=1}^{m}\ell_{i}$ denote the total tree length. Our goal is to determine the expected number of sampled mutations required for every edge of T to receive at least one mutation, ensuring that the tree can be reconstructed. We assume for the present work that the mutation rate is constant and so, if we let $p_{i}$ denote the probability that a mutation lands on edge *i*; given the branch lengths, then these probabilities are proportional to the edge lengths,


(1) $$p_{i}=\frac{\ell_{i}}{L_{\text{tot}}},\;0<p_{i}\leq 1,\;\sum_{i=1}^{m}p_{i}=1.$$

We formalize this as a coverage process over the edges of T. Each mutation independently selects an edge *i* with probability $p_{i}$, and we define the “cover time” random variable *T* as –


(2) $$\begin{array}{c} T=\text{min}\{t\in N:\;\text{every}\;\text{edge}\;i\in E\;\text{has}\;\text{been}\;\text{hit}\;\text{by}\;\text{at}\;\text{least}\;\\ \text{one}\;\text{of}\;\text{the}\;\text{first}\;t\;\text{sampled}\;\text{mutations}\}. \end{array}$$

The quantity of interest throughout the paper is the expected cover time E[T], representing the expected number of mutations required for all edges of T to be observed at least once given tree T. Unless otherwise stated, E[T] refers to this conditional expectation for a fixed tree.

In the following subsections, we divide the analysis into four parts. We begin with the case of a perfect phylogeny with equal branch lengths and derive the exact number of mutations required for complete coverage, showing that it follows the classic O(m log m) behavior. We then establish general lower and upper bounds for the expected cover time when edge lengths are unequal and sampled from an arbitrary distribution. Finally, we extend the analysis to a loss-supported 1-Dollo phylogeny model and derive the corresponding expected number of sampled mutations.

Theorem 1 (Exact cover time for perfect phylogeny with equal branch lengths). Let T be a perfect phylogeny with *m* edges. Let *T* be the number of mutations required until every edge has been hit at least once. Then

(3) $$E[T]=mH_{m},\;H_{m}=\sum_{k=1}^{m}\frac{1}{k}$$

In particular, E[T]=m log m+mγ+O(1) as m→∞, where γ is the Euler–Mascheroni constant.

*Proof.* See proof in the Supplementary Material, available at supplementary material  *Bioinformatics Advances* online. □

We next generalize the problem for unequal edge lengths and thus different probabilities $p_{i}$ of a sampled mutation getting mapped to an edge. In a classic coupon collectors problem, $p_{i}$’s are considered fixed parameters. We generalize this to consider $p_{i}$’s to be random variables following an arbitrary distribution. We will first derive a general upper bound applicable for any such distribution and then consider a special case of the Kingman coalescent with exponentially distributed branch lengths. For the general case, we show in Supplementary Section S1.2, available at supplementary material  *Bioinformatics Advances* online that


(4) $$E[T]=\sum_{Ø\neq S\subseteq [m]}{\left(-1\right)}^{|S|+1}\frac{1}{\sum_{i\in S}p_{i}}=\int_{0}^{\infty }[1-\prod_{i=1}^{m}(1-e^{-p_{i}u})]du.$$

In the equal-probability case $p_{i}=1/m$, the integrand simplifies to $1-{\left(1-e^{-u/m}\right)}^{m}$, and a direct computation gives $E[T]=mH_{m}$ (Supplementary Lemma S3, available at supplementary material  *Bioinformatics Advances* online), where $H_{m}=\sum_{k=1}^{m}1/k$ is the *m*th harmonic number. The following theorem generalizes this result for arbitrary edge probabilities.

Theorem 2 (Universal upper bound). Let $p_{1},\ldots ,p_{m}>0$ with $\sum_{i}p_{i}=1$, and $p_{\text{min}}={\text{min}}_{i}p_{i}=\frac{\ell_{\text{min}}}{L_{\text{tot}}}$. Then

$$E[T]\leq \frac{H_{m}}{p_{\text{min}}},\;H_{m}=\sum_{k=1}^{m}\frac{1}{k}$$

Equality holds if and only if $p_{i}=1/m$ for all edges *i*.

*Proof.* See proof in the Supplementary Material, available at supplementary material  *Bioinformatics Advances* online. □

### 2.1 Consequences

If the edge lengths $\ell_{i}$ are known, $p_{i}=\ell_{i}/L_{\text{tot}}$ and $\ell_{\text{min}}={\text{min}}_{i}\ell_{i}$ give the deterministic bound


$$E[T|T]\leq H_{m}\frac{L_{\text{tot}}}{\ell_{\text{min}}}.$$

### 2.2 Special case: Kingman coalescent trees

The Kingman coalescent (Kingman 1982) is a stochastic model of evolution in a neutrally evolving population of constant size. Going backward in time, lineages randomly merge pairwise until a single common ancestor is reached. When *k* lineages are present, the waiting time to the next coalescent event follows an exponential distribution with rate parameter $\left(\begin{array}{c} k\\ 2 \end{array}\right)$. We explore it as an example of an approximation to the edge length ratios we might expect in more realistic clonal trees. We assume T to be a random rooted Kingman coalescent binary tree on *n* leaves (m=2n−2 edges) whose edge lengths $\ell_{e}$ are independent exponential random variables determined by the aforementioned rates. In this setting, the probabilities $p_{i}=\ell_{i}/L_{\text{tot}}$ are random variables rather than fixed parameters. Taking the expectation over coalescent trees then gives $E[T]\leq H_{m}E_{\ell }[\frac{1}{p_{\text{min}}}]=H_{m}E_{\ell }[\frac{L_{\text{tot}}}{\ell_{\text{min}}}]$. This is the expectation of the ratio of two dependent random variables, which is hard to simplify to a closed-form and often requires knowing the joint distribution (and covariance) of $\ell_{\text{min}}$ and $L_{\text{tot}}$. To obtain a tractable analytic estimate, we use two complementary strategies.

First, we derive a high-probability bound under the assumption that branch lengths $\ell_{1},\ldots ,\ell_{m}$ are independent exponential random variables with rates $\lambda_{1},\ldots ,\lambda_{m}$. For this, we define


$$Y=\ell_{\text{min}}=\underset{i}{\min}\ell_{i},\;J=\text{arg}\underset{i}{\min}\ell_{i},\;\Lambda =\sum_{i=1}^{m}\lambda_{i}$$

The following lemmas, proved in the Supplementary Methods, available at supplementary material  *Bioinformatics Advances* online, characterize the distribution of the minimum branch length and the conditional structure of the remaining edges. With these, we derive a high probability bound for expected cover time conditioned on the minimum branch length in Theorem 3.

Lemma 1 (Minimum index, distribution of the minimum branch length, and conditioning on it). Since $\ell_{i}∼\;\text{Exp}(\lambda_{i})$ are independent,

$$\text{Pr}(J=j)=\frac{\lambda_{j}}{\Lambda },\;Y=\ell_{J}∼\;\text{Exp}(\Lambda ),$$

and (Y,J) has joint density $f_{Y,J}(y,j)=\lambda_{j}e^{-\Lambda y}.$ In particular, *Y* and *J* are independent, and in event {Y=y,J=j}, $\ell_{j}=y$ and $\ell_{i}\geq y$ for all i≠j.

*Proof.* See proof in the Supplementary Material, available at supplementary material  *Bioinformatics Advances* online. □

Lemma 2 (Memoryless decomposition of edge lengths). Conditional on the event {Y=y,J=j}, the minimum–attaining edge satisfies $\ell_{j}=y$, and every other edge length admits the decomposition

$$\ell_{i}=y+A_{i},\;i\neq j,$$

where $A_{i}$’s denote the excess length beyond the minimum and each $A_{i}:=\ell_{i}-y$ is independent of (Y,J) and is distributed as

$$A_{i}∼\;\text{Exp}(\lambda_{i}).$$

Moreover, the residuals $\left\{A_{i}:i\neq j\right\}$ are mutually independent.

*Proof.* See proof in the Supplementary Material, available at supplementary material  *Bioinformatics Advances* online. □

We will also need the mean of the total “excess” length beyond the minimum.

Lemma 3 (Mean excess length). Let $A_{i}$ be as in Lemma 2, and let $X=\sum_{i\neq J}A_{i}.$ Then

$$E[X]=\sum_{i=1}^{m}\frac{1}{\lambda_{i}}-\frac{m}{\Lambda }.$$

*Proof.* See proof in the Supplementary Material, available at supplementary material  *Bioinformatics Advances* online. □

We now prove the conditional cover-time bound with respect to the minimum branch length using Lemmas 1–3.

Theorem 3 (Conditional cover-time bound with respect to the minimum edge). For any x>0,

$$E[T|Y\geq x]\leq H_{m}\left(m+\frac{1}{x}\left(\sum_{i=1}^{m}\frac{1}{\lambda_{i}}-\frac{m}{\Lambda }\right)\right).$$

*Proof.* See proof in the Supplementary Material, available at supplementary material  *Bioinformatics Advances* online. □

Based on Lemmas 1, 2, and Theorem 3, we now prove the high-probability bound for coalescent-insprired exponential branch lengths with Corollary 1.

Corollary 1 (High-probability cover-time bound). Let δ∈(0,1) be a desired failure probability when Y<x and so, Pr(Y≥x)=1−δ. Following this,

$$x=\frac{1}{\Lambda }\text{log}\;\frac{1}{1-\delta }.$$

and on this (1−δ)-probability event,

$$E[T|Y\geq x]\leq H_{m}\left(m+\frac{\Lambda }{\;\text{log}\;\left(1/(1-\delta )\right)}\left(\sum_{i=1}^{m}\frac{1}{\lambda_{i}}-\frac{m}{\Lambda }\right)\right).$$

*Proof.* See proof in Supplementary Material, available at supplementary material  *Bioinformatics Advances* online. □

Although the bounds are somewhat pessimistic, this result implies that, in a tree with independent exponential branch lengths, if the smallest branch length is not too small then, with probability (1−δ), the expected number of mutations required to cover all edges is bounded in terms of δ and the size of the tree. In particular, as long as δ is not extremely small, the number of mutations remains modest for typical sizes of clonal trees. For example, for a 5-clone tree and δ=0.3, Corollary 1 yields E[T∣Y≥x]≤172 mutations needed to resolve the tree correctly.

Second, we consider a deterministic approximation to the true coalescent in which each coalescent epoch has length fixed to its expected value. In this mean-epoch model, the time between the coalescent events for *k* lineages is $1/\left(\begin{array}{c} k\\ 2 \end{array}\right)$. The minimum edge length therefore equals $\ell_{\text{min}}=\frac{1}{\left(\begin{array}{c} n\\ 2 \end{array}\right)}$ and the total expected tree length is $L_{\text{tot}}=2H_{n-1}$, as we prove in Supplementary Lemma S4, available at supplementary material  *Bioinformatics Advances* online. Applying Theorem 2 with $p_{\text{min}}=\ell_{\text{min}}/L_{\text{tot}}$ yields


(5) $$E[T]\leq \frac{H_{m}}{p_{\text{min}}}=H_{m}\frac{L_{\text{tot}}}{\ell_{\text{min}}}=H_{m}(2H_{n-1})\left(\begin{array}{c} n\\ 2 \end{array}\right)=n(n-1)H_{m}H_{n-1}$$

Since $H_{n}=\Theta (\text{log}\;n)$, this leads to an asymptotic upper bound of $O(n^{2}{\left(\text{log}\;n\right)}^{2})$. Thus, under this deterministic mean-epoch approximation, the expected number of mutations required to cover all branches of a coalescent tree scales quadratically with the number of lineages, up to logarithmic factors.

Next, we extend the analysis to evolutionary models that permit mutation losses. In cancer evolution, gained mutations are frequently lost due to independent events or through larger-scale copy number deletions (Kuipers *et al*. 2017). To account for this, we move beyond the classical infinite-sites model and use the *K*-Dollo model, which assumes that each mutation may be gained once but can be lost up to *K* times along the phylogeny. The perfect phylogeny corresponds to the special case K=0. In our analysis of the *K*-Dollo models, we continue to assume equal branch lengths, as in the perfect-phylogeny setting. We derive the exact expected number of sampled mutations required for full edge coverage under the *1-Dollo model* (K=1), leaving the general *K*-Dollo case as a direction for future work.

Theorem 4 (Expected cover time under a simplified 1-Dollo model). Under the 1-Dollo assumption, each mutation is gained once and may be lost at most once on a descendant branch of the gain. A single mutation can therefore cover either one previously uncovered edge (gain-only) or two uncovered edges (gain–loss). Although the tree-constrained 1-Dollo model requires the loss edge to be descendant from the gain edge, for analytical tractability we derive the following bound under a simplified independent coverage model, in which the gain and loss edges are treated as independently selected edges. Let $p=P_{\text{loss}}\in [0,1)$ denote the probability that a gain undergoes loss. Then the expected number of mutations required to cover all *m* edges is

(6) $$E[T]=\frac{m}{1+p}\left(H_{m}-\sum_{k=1}^{m}\frac{{\left(-p\right)}^{m-k+1}}{k}\right),\;H_{m}=\sum_{k=1}^{m}\frac{1}{k}$$

*Proof.* See proof in Supplementary Material, available at supplementary material  *Bioinformatics Advances* online. □

Corollary 2 (No-loss case, i.e. perfect phylogeny). For P=0 (mutations gained once and never lost), the inner sum vanishes and

$$E[T]=mH_{m},$$

recovering the classical coupon-collector expectation.

### 2.3 Code availability

All scripts used for simulation are implemented in Python 3 and available at https://github.com/CMUSchwartzLab/mutation-subsampling.git.

## 3 Experimental results

We have conducted experiments on simulated and real data designed to validate the correctness of the bounds shown in this paper, assess how pessimistic they are in practice, and test how well they extend to more complex but realistic evolutionary scenarios beyond those covered in the proofs. We first validated the robustness of the bounds using simulated data and assessed tree reconstruction accuracies with respect to SPhyR (El-Kebir 2018) and PAUP (Wilgenbusch and Swofford 2003) for perfect and 1-Dollo phylogenies, and with PAUP for coalescent phylogenies. We further used simulated data to assess the k-Dollo case and the full coalescent model empirically. We also evaluated the bounds on real cancer samples, limiting comparisons to SNVs as in our theoretical bounds, although the true data contain SNVs, CNAs, and SVs. The Supplementary Results, available at supplementary material  *Bioinformatics Advances* online show further testing on simulated data and a high-grade serous ovarian cancer (Funnell *et al*. 2022).

### 3.1 Evaluation criteria

To assess the quality of the estimated tree topologies on simulated datasets, we compared them with ground-truth trees using *normalized Robinson-Foulds (RF) distance* (Robinson and Foulds 1981). To evaluate branch-length accuracy, especially under mutation subsampling, we further computed the *mean absolute error (MAE)* between the true versus estimated branch lengths after normalizing the branch lengths for each tree by that tree’s total branch length, computed over all pairs of leaves in the *n*-leaf trees. Additionally, to evaluate the accuracy of mutation placement on the inferred trees, we compute the *pairwise ancestral relationship accuracy* as described in Supplementary Section S2.1, available at supplementary material  *Bioinformatics Advances* online.

### 3.2 Evaluation on simulated data

To evaluate how much phylogeny quality is affected by mutation counts across the range from the lower and upper bounds established theoretically, we simulated single-cell WGS data with SNVs based on known ground-truth tumor and coalescent phylogenies. We explain the simulation process for perfect, *K*-Dollo and coalescent phylogenies in detail in the following sections.

### 3.3 Perfect and 1-Dollo phylogeny simulations

**Figure 1:**
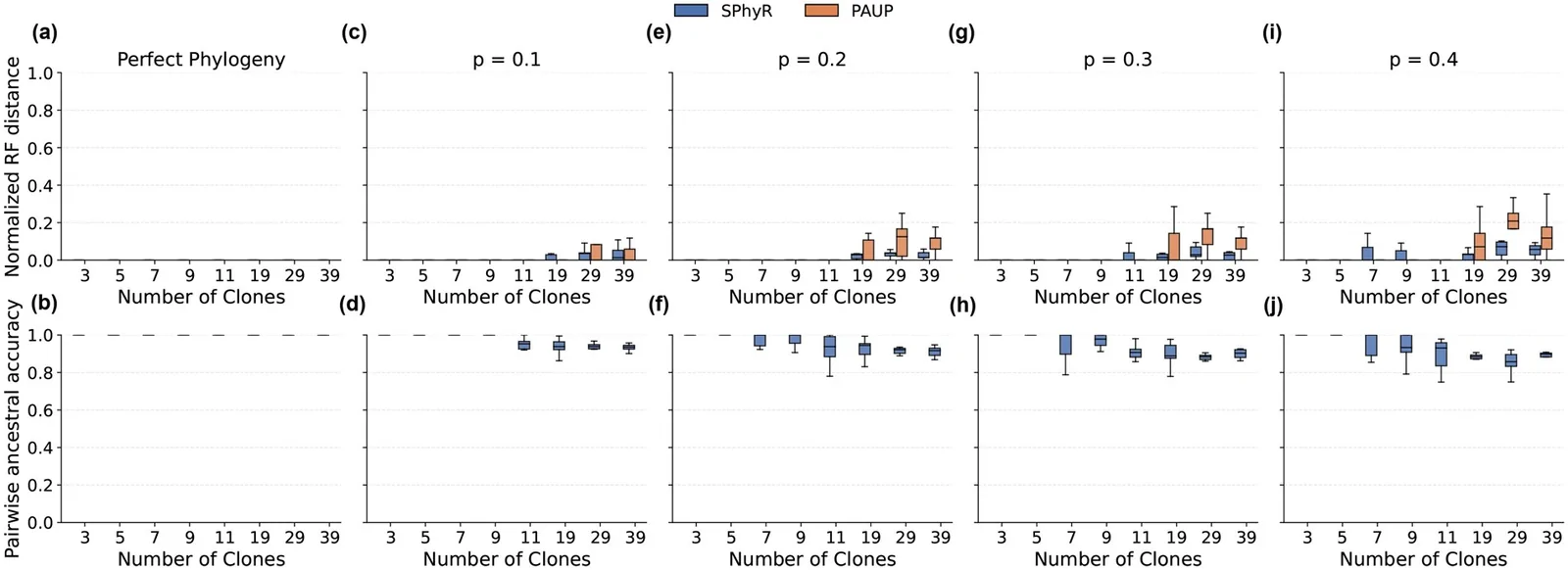
Results for perfect and 1-Dollo phylogeny reconstruction with SPhyR, where mutations are sampled using Equations 3 and 6 respectively for different number of clones N∈{3,5,7,9,11,19,29,39}. For the 1-Dollo phylogenies, we consider different loss probabilities, p∈{0.1,0.2,0.3,0.4}.

To evaluate the robustness of our bounds for perfect and 1-Dollo simulations, we first generated random full binary perfect phylogenies with n∈{2,3,4,5,6,10,15,20} leaves, to span a range typical of clonal trees in cancers. This corresponds to (2n−1)∈{3,5,7,9,11,19,29,39} total nodes or clones in the tree. Since a full binary tree with *n* leaves has m=2n−2 edges, we generated $mH_{m}$ mutations and mapped each of these $mH_{m}$ mutations to a uniformly chosen edge for each full binary perfect phylogeny, $T^{*}$. After assigning all mutations, we counted how many branches of the perfect phylogeny remained without any mutation. We simulated 10 replicates for each value of *n*. Figure S2, available at supplementary material  *Bioinformatics Advances* online shows the distribution of the number of unmapped branches. The results indicate that using the perfect-phylogeny bound almost always results in every branch receiving at least one mutation. We ran SPhyR (El-Kebir 2018) on the generated perfect phylogenies with $mH_{m}$ mutations. Figure 1 shows the results of SPhyR for perfect phylogenies with the aforementioned number of clones. In each case, we reconstruct the ground truth tree with SPhyR, resulting in zero normalized RF distance and pairwise ancestral accuracies = 1.

To generate 1-Dollo phylogenies, we extended each perfect phylogeny $T^{*}$ with $mH_{m}$ mutations by introducing single-loss events. For each mutation, we independently introduced a loss with probability *p*, removing the mutation and all of its descendants. Loss placement is determined by a depth-first traversal of the tree. At each internal branch, we consider mutations that are currently present on that branch, were not gained on that same branch, and still have remaining allowed losses. Each such mutation is then assigned a loss independently with probability *p*. When a loss occurs on a branch, the mutation is removed from all descendants in that subtree. This produces 1-Dollo instances that contain both gains and single independent losses while preserving the structure of $T^{*}$. We then sample a subset of mutations according to Equation (S4, available at supplementary material  *Bioinformatics Advances* online), run SPhyR (El-Kebir 2018) and PAUP (Wilgenbusch and Swofford 2003) on the sampled data, and compare the reconstructed trees against the full 1-Dollo phylogeny. Figure 1 shows the 1-Dollo phylogeny reconstruction accuracies. We run 10 replicates for each loss probability p∈{0.1,0.2,0.3,0.4} and vary the number of clones as {3,5,7,9,11,19,29,39}. For each simulated instance, we run SPhyR (El-Kebir 2018) and compare the reconstructed trees using normalized Robinson–Foulds (RF) distances (top panel) and pairwise ancestral relationship accuracies (bottom panel). As the number of clones increases, the normalized RF distance increases slightly, although the reconstructed trees remain highly accurate. We see similar results when evaluating mutation placements via pairwise ancestral relationship accuracy. With three and five clones, there is little difference between the sampled and full 1-Dollo trees, supporting the accuracy of the reconstructed trees for both cases. However, as the number of clones increases, the gap between the mutations in the full and sampled 1-Dollo trees widens, increasing the complexity for SPhyR and leading to larger reconstruction errors. We see similar results for different values of the mutation loss probability, p∈{0.1,0.2,0.3,0.4}. Since PAUP does not have a dedicated 1-Dollo setting and instead attempts to reconstruct the tree under a perfect-phylogeny-like model, so its accuracy decreases as *p* increases because the data contain more losses while the number of sampled mutations is also reduced according to Equation (6). By contrast, SPhyR is designed to accommodate mutation losses, and therefore the same monotone decrease is not evident from the empirical results. Overall, although higher loss probabilities result in fewer sampled mutations and make inference more challenging, both topology and phylogenetic placement remain highly accurate on the ranges of clones considered, well beyond the roughly 5–10 clones typically resolved in tumor phylogeny studies.

### 3.4 Empirical evaluation on *k*-Dollo phylogenies

We generalize beyond the theoretical bounds of the 1-Dollo phylogenies to a more realistic scenario for somatic evolutionary trees, simulating *K*-Dollo phylogenies following SPhyR (El-Kebir 2018) and the similar method as described in the previous section, by introducing losses in the perfect phylogeny, $T^{*}$ for each mutation with loss probability *p*, allowing at most *k* losses per mutation. Specifically, we iteratively select a mutation and introduce a loss with probability *p* if the mutation currently has at most (k−1) losses. Once a loss is introduced for mutation *i*, all descendants of the corresponding node are set to carry that loss.

**Figure 2:**
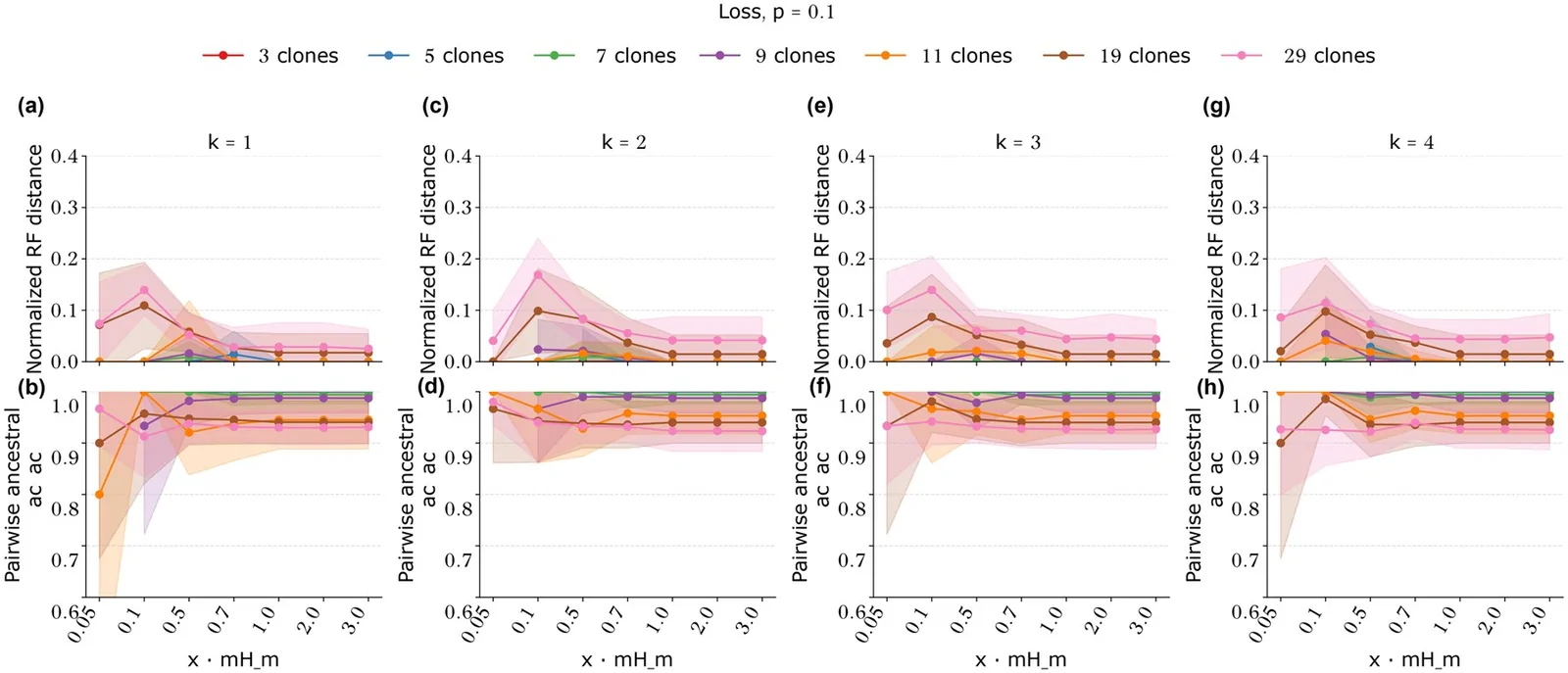
Results for *k*-Dollo phylogeny reconstruction with SPhyR (El-Kebir 2018). The figure shows normalized phylogeny inference (top) and pairwise ancestral accuracy (bottom) across a range of *k*, clone numbers, and sampling rates for loss probability, P=0.1.

Since we do not derive an analytical bound for *k*-Dollo phylogenies, we instead provide empirical results for k∈{1,2,3,4}. For each *k*, we vary the number of sampled mutations as a multiple *x* of $mH_{m}$ ($x\times mH_{m}$), where x∈{0.05,0.1,0.5,0.7,1,2,3}, where x=1 corresponds to exactly the number of mutations needed according to the perfect phylogeny bound and *x* below or above one corresponds to mutations below or in excess of that bound, respectively. We then run SPhyR on the simulated instances with its *K*-Dollo setting. Figure 2 summarizes the empirical estimates. To make the comparison fair, we remove from the full tree the mutations that were not sampled, contract any edges left without mutations, and then compute the RF distance and pairwise ancestral accuracy on the resulting reduced trees. In the very low-coverage settings, such as 0.05× and 0.1×, only a small number of mutations survive, and thus only a very small number of edges remain in the comparison. This can make the inferred tree appear artificially accurate at 0.05×, since the comparison is being made on a much simpler reduced structure. At 0.1×, however, there are more mutations to introduce additional structural complexity, but still not enough to reconstruct the reduced tree reliably, leading to a temporary drop in accuracy. By 0.5×, the number of sampled mutations is closer to being sufficient, and reconstruction accuracy improves again. Across all *k* values, we observe that the normalized RF distances decrease and the pairwise ancestral relationship accuracies increase beginning around x=0.7 – 1.0, indicating that the trees become reliably reconstructable once the number of sampled mutations reaches this range for a loss probability of P=0.1. When we calculated the theoretical ratio for x by taking the ratio of the thoeretical bounds for 1-Dollo and perfect phylogeny models, we derived the values {1.0,0.89,0.93,0.91,0.9,0.92,0.92} respectively for clones ∈{3,5,7,9,11,19,29}, which matches the empirical results from the k=1 case. We additionally simulated *k*-Dollo phylogenies with higher loss probabilities p∈{0.2,0.3,0.4}. In the supplementary figures (Figs S3–S5, available at supplementary material  *Bioinformatics Advances* online), we observe similar trends to the p=0.1 case. However, as both the loss probability and *k* increase, SPhyR requires larger number of sampled mutations to infer accurate phylogenies.

### 3.5 Coalescent tree simulations

To simulate coalescent trees with *n* leaves representing *n* observed clones, we use CellCoal (Posada 2020), which starts by simulating a genealogy for the sampled cells under the neutral coalescent model with an exponential population growth model (Slatkin and Hudson 1991) for a defined parameterization of the coalescent for cancer cell samples (Ohtsuki and Innan 2017). A healthy somatic cell is considered to define the “outgroup branch” for the cell phylogeny. We simulate 10 replicates for each of n∈{3,5,7,9,11} clone trees, running CellCoal with 0/1 alphabet under the infinite sites model (ISM).

We considered two settings of CellCoal. (1) In the first setting, we explicitly defined the coalescent parameters, setting the effective population size to $10^{5}$, the exponential growth rate to $10^{-5}$ and 10 000 variant sites, and generated trees whose branch lengths were exponential random variables following the Kingman Coalescent model. (2) In the second setting, we generated trees with branch lengths fixed to the expected mean-epoch lengths under the coalescent model, such that each inter-coalescent interval corresponded exactly to its theoretical mean. For both settings, the simulated healthy outgroup cell is generated from a reference genome and includes representative germline SNP positions, which we exclude from downstream analyses to ensure only somatic variants are considered.

After the simulation, we run PAUP (Wilgenbusch and Swofford 2003) to reconstruct phylogenies from the coalescent-simulated data. To test how phylogeny reconstruction performs with different numbers of sampled mutations, we first run PAUP with all the non-SNP positions from the coalescent simulations. We then repeat the analysis with $n(n-1)H_{m}H_{n-1}$ subsampled mutations, following the mean-epoch coalescent bound in Equation (5).

**Figure 3:**
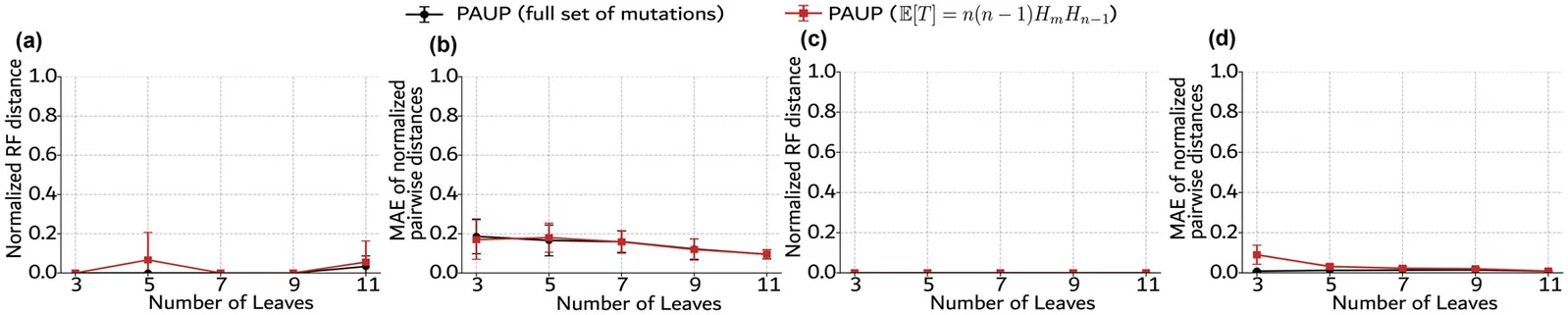
Results of coalescent phylogeny reconstruction using the full set (black lines) and subsampled set (red lines) of mutations across varying numbers of leaves, n∈3,5,7,9,11. (a) And (b) show the normalized Robinson–Foulds (RF) distance and the mean absolute error (MAE) in branch-length estimation for random coalescent simulations following the Kingman model. (c) And (d) show the corresponding RF distance and MAE for coalescent simulations with branch lengths fixed to the expected mean-epoch lengths under the coalescent model.

Figure 3a and b show the normalized Robinson–Foulds (RF) distance and the mean absolute error (MAE) of normalized branch lengths for the random coalescent-branch simulations, while Fig. 3c and d show the corresponding results for trees with deterministic mean-epoch branch lengths. In both cases, black lines represent tree reconstructions using the full set of mutations, whereas red lines correspond to subsampling exactly $n(n-1)H_{m}H_{n-1}$ mutations. We see that across both simulation models, tree topologies were reconstructed with high accuracies even under mutation subsampling, indicating that the predicted number of mutations is sufficient to recover the correct tree structures. However, the branch-length estimation was more challenging for trees with random coalescent branch lengths, with PAUP exhibiting higher variability and error, whereas trees with deterministic mean-epoch lengths showed substantially more consistent and accurate branch-length estimation.

### 3.6 Evaluation on a metastatic colorectal cancer patient

**Figure 4:**
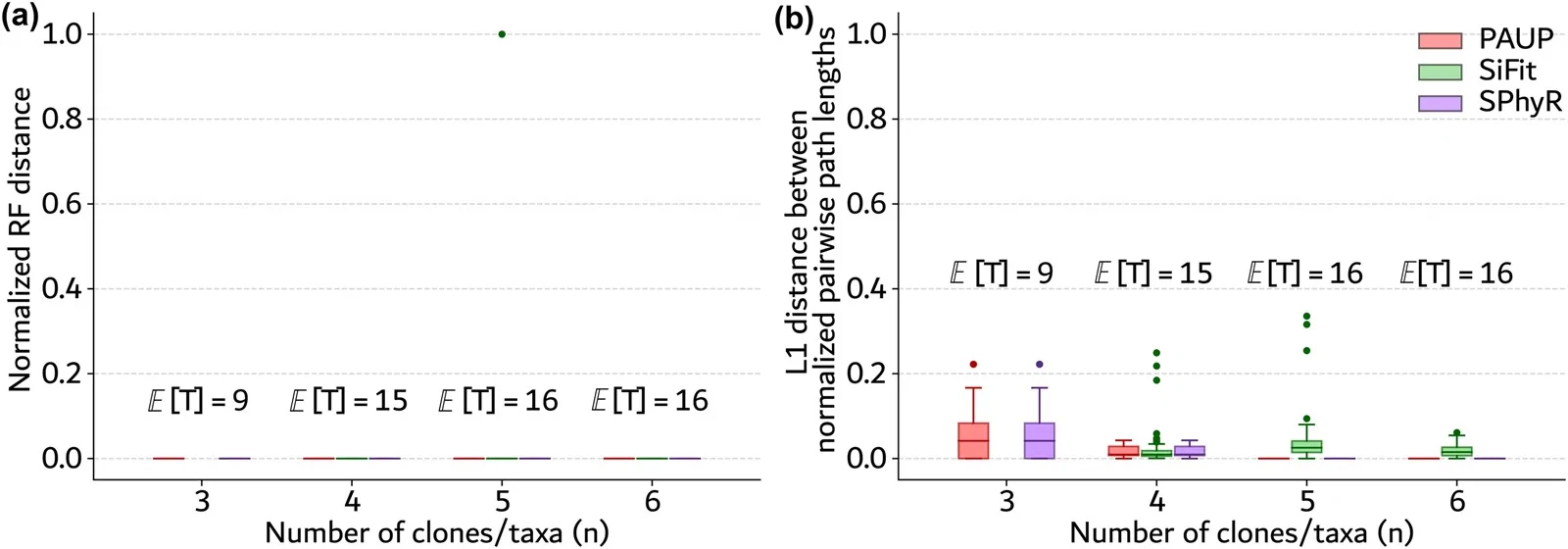
Results on the CRC1 patient dataset (Leung *et al*. 2017) for n∈3,4,5,6 taxa (clusters). We ran PAUP (Wilgenbusch and Swofford 2003) using the neighbor-joining method for 10 replicates of the sampled mutations with E[T]∈9,15,16,16 for n∈3,4,5,6, respectively.

To evaluate the robustness of our theoretical bounds on a real cancer dataset, we analyzed the metastatic colorectal cancer dataset (CRC1) from Leung *et al*. (2017), which includes 178 single cells and 16 single-nucleotide variants (SNVs) identified through targeted sequencing of 1000 cancer-associated genes. Given the number of mutations available, we considered reconstruction of trees assuming 3–6 clusters to allow for cases where the number of SNVs is in excess of the theoretical bound (3–4 clusters) and where the bound suggests the data will be too limited (5–6 clusters). We clustered the cells with *K*-means clustering and randomly selected one representative from each cluster. Since the theoretical bounds exceeded the number of SNVs available for reconstructing a perfect phylogeny with more than four clusters, we tested 10 replicates of three and four taxa trees, comparing reconstructions from the sampled set of mutations versus the full set of mutations. We additionally restricted the number of mutations to the maximum available in the dataset and reconstructed five and six cluster trees using PAUP (Wilgenbusch and Swofford 2003). In each case, PAUP produced identical trees from the sampled versus full mutation sets, resulting in a normalized Robinson–Foulds (RF) distance of 0. To further assess similarity, we compared the L1 distance of the pairwise branch lengths normalized by total tree length for the full and sampled clonal trees. As shown in Fig. 4, the number of sampled mutations increases with the number of taxa, while the L1 distance between the branch-length ratios of the full and sampled trees decreases, indicating increasing structural similarity. The results confirm that following the theoretical bounds reliably yields equivalent topologies to the full data set, although with some error in inferred edge lengths.

## 4 Discussion

This paper examined the problem of how many mutations one needs to reliably reconstruct clonal lineage trees from SNV data under various evolutionary models, extending prior theory from species evolution (Erdos *et al*. 1999) to models more relevant to somatic evolution. The question is motivated by a practical problem of how to solve for complex and computationally intensive evolutionary models needed in somatic clone tree applications in the face of increasingly large variation data sets. We established theoretical bounds for some simplified evolutionary models commonly used in clonal lineage studies. These bounds tend to be more pessimistic than those in the seminal work of Erdos *et al*. (1999) on a more simplified model, but they nonetheless show that modest numbers of variants are sufficient to derive the correct tree topologies for realistic numbers of clones. Empirical studies with simulated and real tumor data confirm that these bounds provide good guidance in practice despite the simplified evolutionary models from which they derive.

This work provides a firmer basis for a practical strategy of dealing with large variant sets and algorithms that scale poorly in their size by subsampling to smaller numbers of variants to derive the correct tree followed by using faster algorithms to place the remaining variants on the tree. Considerable room remains, though, to extend the work further. The theory so far covers only some relatively simplified evolutionary models typical of those assumed in somatic phylogeny methods, but might be extended to more involved models that better capture the true biology, for example in allowing arbitrary mutation gain/loss, more comprehensive variant types, variable mutation rates, or accounting explicitly for erroneous and missing data. Another important direction is to study additional evolutionary regimes, such as non-neutral evolution, selective sweeps, bottlenecks, and more general finite-sites models with recurrent mutation gains and reversions. Results on simulated and real data suggest that the bounds we establish hold at least approximately for more realistic data. The present work has only looked at SNV data, and both the theoretical and empirical studies could be extended to cover the harder cases of evolution by CNA and SV events important in somatic evolution processes. There is likely also room for improvement in algorithms for both inference of topologies from small variant sets and on fast placement of unsampled variants on those topologies, as well as other extensions to more effectively use unsampled variants to validate topologies or assess uncertainty within them. Another direction for future work is to complement these sufficiency results with characterizations of the minimum amount of data required for reconstruction. Furthermore, there may be other uses of the derived bounds to explore. For example, while we focused on the scenario of how to subsample mutations when they are available in excess, these bounds also apply to a parallel problem we touched on only briefly of how to define the finest resolution of clonal structure a data set can support, for example the maximum number of clones or the shortest edge lengths it can reliably resolve, when mutation data is sparse.

## Supplementary Material

vbag264_Supplementary_Data

## Data Availability

The data underlying this article are available in the article, its online supplementary material, and its associated code repository https://github.com/CMUSchwartzLab/mutation-subsampling.git.
