# Supplementary material for: Theoretical estimates on the expected number of mutations needed to reconstruct clonal lineage trees
Source: Bioinform Adv. 2026 Sep 7;6(1):vbag264. doi: 10.1093/bioadv/vbag264 (PMC13614416; doi:10.1093/bioadv/vbag264)
Supplement: Supplementary file 1 [file vbag264_supplementary_data.pdf]

Supplementary Material for Bristy and Schwartz – Theoretical estimates on the expected number of mutations needed to reconstruct clonal lineage trees

We include supplementary materials to provide additional detail omitted from the main paper due to space limitations. Supplementary Methods is devoted to theoretical derivations and proofs for results cited in the main paper. Supplementary Results provides details on additional simulation tests supporting the conclusions of the main paper.

Supplementary Methods

Cover Time Bounds for Perfect Phylogeny

Proof of Theorem 1: Exact cover time for perfect phylogeny with equal branch lengths

Theorem 1 (Exact cover time for perfect phylogeny with equal branch lengths)**.** *Let $\mathcal{T}$ be a perfect phylogeny with m edges. Let T be the number of mutations required until every edge has been hit at least once. Then*

$$\mathbb{E}[T] = mH_m, \qquad H_m = \sum_{k=1}^{m} \frac{1}{k} \tag{3}$$

In particular, $\mathbb{E}[T] = m \log m + m\gamma + O(1)$ as $m \to \infty$, where γ is the Euler–Mascheroni constant.

Proof. Each sampled mutation falls on one of the m edges with probability $\frac{1}{m}$. With $p_i = 1/m$, the process of assigning mutations to edges is the classical coupon collector with m equiprobable coupons. Let T_i denote the number of additional mutations needed to discover a new edge given that $i-1$ distinct edges have already been seen ($i = 1, \ldots, m$). At that time, the success probability for hitting a previously unseen edge is $q_i = \frac{m-(i-1)}{m}$ Hence T_i is geometric with mean $\mathbb{E}[T_i] = \frac{1}{q_i} = \frac{m}{m-(i-1)}$. Since $T = \sum_i T_i$, linearity of expectation gives $\mathbb{E}[T] = \sum_{i=1}^{m} \mathbb{E}[T_i]$.

$$\mathbb{E}[T] = \sum_{i=1}^{m} \mathbb{E}[T_i] = \sum_{i=1}^{m} \frac{1}{q_i} = \sum_{i=1}^{m} \frac{m}{m-(i-1)} = m \sum_{i=1}^{m} \frac{1}{i} = mH_m$$

where H_m is the mth harmonic number. This can be approximated as $\mathbb{E}[T] = m \log m + m\gamma + \frac{1}{2} + O(\frac{1}{m}) = m \log m + m\gamma + O(1)$, where $\gamma \approx 0.577215$ is the Eular-Mashcheroni's constant. For large m, $\mathbb{E}[T] \approx m \log m + m\gamma$. □

Derivation of the Integral Identity for $\mathbb{E}[T]$

Theorem S1 (Integral Identity for Expected Cover Time under Nonuniform Branch Probabilities)**.** *Let T denote the number of independent mutation events required until every edge of a phylogenetic tree with m edges has received at least one mutation. Assuming that each mutation independently targets edge i with probability $p_i > 0$, where $\sum_{i=1}^{m} p_i = 1$. Then the expected cover time $\mathbb{E}[T]$ admits the integral representation*

$$\mathbb{E}[T] = \int_0^\infty \left[1 - \prod_{i=1}^{m} \left(1 - e^{-p_i u}\right)\right] du \tag{S1}$$

Proof. We sketch the derivation, following a generalized coupon–collector like argument for nonuniform sampling probabilities. For any nonnegative integer-valued random variable T, let $\Pr(T > t)$ is the probability that after t mutations at least one edge remains unmapped. Thus we have:

$$\mathbb{E}[T] = \sum_{t \geq 0} \Pr(T > t) \tag{S2}$$

Let $A_i(t)$ denote the event that edge i has not been hit by any of the first t mutations. Then the event $\{T > t\}$ occurs when at least one such event holds. Therefore, $\{T > t\} = \bigcup_{i=1}^{m} A_i(t)$. Because these events are not disjoint, since multiple edges may remain unmutated simultaneously, we calculate the probability of this union with the inclusion-exclusion principle:

$$\Pr(T > t) = \sum_{\emptyset \neq S \subseteq [m]} (-1)^{|S|+1} \Pr\Big(\bigcap_{i \in S} A_i(t)\Big)$$

The term with $|S| = 1$ adds the probability that a single edge remains unmutated, the terms with $|S| = 2$ subtract the probability that two edges remain unmutated simultaneously (since these were double-counted), and so on. Thus the alternating sum from the inclusion-exclusion principle exactly corrects for all overlaps among the events $A_i(t)$.

To evaluate $\Pr(\bigcap_{i \in S} A_i(t))$, let's consider one mutation event. Each mutation independently targets edge i with probability p_i, where $\sum_{i=1}^{m} p_i = 1$. Hence, for a single mutation, the probability that it *does not* hit any edge in S is

$$1 - \sum_{i \in S} p_i,$$

since the total probability of hitting any edge in S is $\sum_{i \in S} p_i$. Because the t mutations are independent, the probability that all t of them avoid every edge in S is obtained by multiplying this probability across all t draws:

$$\Pr\Big(\bigcap_{i \in S} A_i(t)\Big) = \big(1 - \sum_{i \in S} p_i\big)^t$$

Intuitively, this is the probability that none of the t mutations falls on any of the edges in S, i.e., that all edges in S remain unmutated after t mutation events. Applying this to Equation (S2), we get

$$\begin{aligned}\mathbb{E}[T] &= \sum_{t=0}^{\infty} \Pr(T > t) \\ &= \sum_{\emptyset \neq S \subseteq [m]} (-1)^{|S|+1} \sum_{t=0}^{\infty} \Big(1 - \sum_{i \in S} p_i\Big)^t \\ &= \sum_{\emptyset \neq S \subseteq [m]} (-1)^{|S|+1} \frac{1}{\sum_{i \in S} p_i} && \text{(geometric series with ratio } 1 - \sum_{i \in S} p_i \in [0,1))\end{aligned}$$

Finally, using $\frac{1}{x} = \int_0^{\infty} e^{-xu} du$ for $x = \sum_{i \in S} p_i$, the inclusion-exclusion identity $\prod_{i=1}^{m}(1 - z_i) = \sum_{S \subseteq [m]} (-1)^{|S|} \prod_{i \in S} z_i$, and substituting $z_i = e^{-p_i u}$ gives:

$$\mathbb{E}[T] = \int_0^{\infty} \Big[1 - \prod_{i=1}^{m} (1 - e^{-p_i u})\Big] du,$$

which is Equation (4) in the main text. □

Lemma S4 (Equal branch lengths)**.** *If $p_i = \frac{1}{m}$ for all $i \in [m]$, then*

$$\mathbb{E}[T] = \int_0^{\infty} \Big[1 - (1 - e^{-u/m})^m\Big] du = mH_m$$

Proof.

$$\begin{aligned}\mathbb{E}[T] &= \int_0^{\infty} \Big[1 - (1 - e^{-u/m})^m\Big]\, du && \text{(from Equation (S1))} \\ &= m \int_1^0 \frac{1 - (1-x)^m}{x} (-dx) && (x = e^{-u/m}, \text{ so } du = -\tfrac{m}{x} dx,\ u: 0 \to \infty \Rightarrow x: 1 \to 0) \\ &= m \int_0^1 \frac{1 - (1-x)^m}{x} dx && \text{(flipping limits to remove the minus)} \\ &= m \int_0^1 \sum_{k=0}^{m-1} (1-x)^k dx = m \sum_{k=0}^{m-1} \frac{1}{k+1} = mH_m && \text{(geometric series; integrating } (1-x)^k \text{ on } [0,1])\end{aligned}$$

□

Lemma S5 (Expected total branch length of the Kingman coalescent trees)**.** *Let L_{tot} be the total tree length (sum of all branch lengths) of the Kingman coalescent tree with n leaves and rate $\binom{k}{2}$ when there are k lineages. Then*

$$\mathbb{E}[L_{tot}] \;=\; 2 \sum_{j=1}^{n-1} \frac{1}{j} \;=\; 2\, H_{n-1}$$

Proof. Let T_k be the waiting time until next coalescence, when k lineages are present. The total tree length is the sum of the branch lengths contributed by each coalescent event:

$$L_{\text{tot}} = \sum_{k=2}^{n} k\, T_k$$

Under the coalescent model, T_k follows an exponential distribution with rate $\binom{k}{2}$, hence $\mathbb{E}[T_k] = 1/\binom{k}{2}$ Taking expectations gives:

$$\mathbb{E}[L_{\text{tot}}] = \sum_{k=2}^{n} k\, \mathbb{E}[T_k] = \sum_{k=2}^{n} \frac{k}{\binom{k}{2}} = \sum_{k=2}^{n} \frac{2}{k-1} = 2 \sum_{j=1}^{n-1} \frac{1}{j} = 2\, H_{n-1}$$

□

Universal Upper Bound on Cover Time

Proof of Theorem 2: Universal Upper Bound

Theorem 2 (Universal upper bound)**.** *Let $p_1, \ldots, p_m > 0$ with $\sum_i p_i = 1$, and $p_{\min} = \min_i p_i = \frac{\ell_{\min}}{L_{tot}}$. Then*

$$\mathbb{E}[T] \;\leq\; \frac{H_m}{p_{\min}}, \qquad H_m = \sum_{k=1}^{m} \frac{1}{k}$$

Equality holds if and only if $p_i = 1/m$ for all edges i.

Proof. Starting from Equation (4), note that $x \mapsto 1 - e^{-xu}$ is increasing in x, so $1 - e^{-p_i u} \geq 1 - e^{-p_{\min} u}$ for all i. All factors lie in $(0, 1]$, hence

$$\prod_{i=1}^{m}(1 - e^{-p_i u}) \geq (1 - e^{-p_{\min} u})^m$$
$$\Rightarrow \quad 1 - \prod_{i=1}^{m}(1 - e^{-p_i u}) \leq 1 - (1 - e^{-p_{\min} u})^m$$

Integrating both sides over $u \in [0, \infty)$ and substituting $v = p_{\min} u$ yields

$$\begin{aligned}
\mathbb{E}[T] &\leq \int_0^\infty \left[1 - (1 - e^{-p_{\min} u})^m\right] du \leq \frac{1}{p_{\min}} \int_0^\infty \left[1 - (1 - e^{-v})^m\right] dv \\
&\leq \frac{1}{p_{\min}} \int_1^0 \frac{1 - (1 - x)^m}{-x} dx \\
&\qquad (\text{Taking } x = e^{-v}, dv = \frac{dx}{-x}) \\
&\leq \frac{1}{p_{\min}} \int_0^1 \sum_{k=0}^{m-1} (1 - x)^k dx \leq \frac{1}{p_{\min}} \sum_{k=0}^{m-1} \int_0^1 (1 - x)^k dx \\
&\leq \frac{1}{p_{\min}} \sum_{k=0}^{m-1} \frac{-1}{k+1} [1 - x]_0^1 \leq \frac{1}{p_{\min}} \sum_{k=1}^{m} \frac{1}{k} \leq \frac{H_m}{p_{\min}}
\end{aligned}$$

Equality holds only if $p_i = p_{\min} = \frac{1}{m}$ for all i. □

High-Probability Bounds under Different Models

Corollary S3 (High-probability cover-time bound for perfect phylogeny with equal branch lengths). *For any $t > 0$,*

$$\Pr(T > t) \leq m e^{-t/m}.$$

Proof. For each branch $i \in [m]$, let A_i denote the event that branch i is not hit in the first t sampled mutations. Then

$$\{T > t\} = \bigcup_{i=1}^{m} A_i.$$

Since each mutation lands uniformly on one of the m branches, the probability that a fixed branch is missed by a single mutation is $1 - \frac{1}{m}$. By independence, for each i, $\Pr(A_i) = \left(1 - \frac{1}{m}\right)^t$. Applying the union bound gives

$$\Pr(T > t) = \Pr\left(\bigcup_{i=1}^{m} A_i\right) \leq \sum_{i=1}^{m} \Pr(A_i) = m\left(1 - \frac{1}{m}\right)^t.$$

Using the inequality $1 - x \leq e^{-x}$ for $x \geq 0$, with $x = \frac{1}{m}$, we obtain $\left(1 - \frac{1}{m}\right)^t \leq e^{-t/m}$. Therefore,

$$\Pr(T > t) \leq m e^{-t/m}.$$

This bound is asymptotically sharper than the Markov bound, $\Pr(T > t) \leq \frac{\mathbb{E}[T]}{t} \leq \frac{m \log m + \gamma + O(1)}{t}$, since it decays exponentially in t rather than only inversely in t. □

A coalescent-inspired conditional cover-time bound conditioned on the minimum branch length

We now discuss a case where the branch lengths, $\ell_1, \ldots, \ell_m$ are assumed to be independent exponential random variables with rates $\lambda_1, \ldots, \lambda_m$, and define

$$Y = \ell_{\min} = \min_i \ell_i, \qquad J = \arg\min_i \ell_i, \qquad \Lambda = \sum_{i=1}^{m} \lambda_i$$

We will prove the following lemma to derive a high probability bound for expected cover time conditioned on the minimum branch length.

Proof of Lemma 1: Minimum index, distribution of the minimum branch length, and conditioning on it

Lemma 1 (Minimum index, distribution of the minimum branch length, and conditioning on it). *Since $\ell_i \sim \mathrm{Exp}(\lambda_i)$ are independent,*

$$\Pr(J = j) = \frac{\lambda_j}{\Lambda}, \qquad Y = \ell_J \sim \mathrm{Exp}(\Lambda),$$

and (Y, J) has joint density $f_{Y,J}(y, j) = \lambda_j e^{-\Lambda y}$. In particular, Y and J are independent, and in event $\{Y = y, J = j\}$, $\ell_j = y$ and $\ell_i \geq y$ for all $i \neq j$.

Proof. For independent exponentials, the event $\{Y > y\}$ means $\ell_i > y$ for all i, hence

$$\begin{aligned}
\Pr(Y > y) &= \Pr(\ell_1 > y, \ldots, \ell_m > y) \\
&= \prod_{i=1}^{m} \Pr(\ell_i > y) \\
&= \prod_{i=1}^{m} e^{-\lambda_i y} \\
&= e^{-(\sum_i \lambda_i) y} \\
&= e^{-\Lambda y},
\end{aligned}$$

so $Y \sim \mathrm{Exp}(\Lambda)$.

To obtain the joint distribution of (Y, J), note that the event $\{Y \in [y, y + dy], J = j\}$ corresponds to edge j taking value in $[y, y + dy]$ while all other edges exceed y. Thus, using independence and the density of ℓ_j,

$$\Pr\big(Y \in [y, y + dy],\, J = j\big) = f_{\ell_j}(y) dy \cdot \prod_{i \neq j} \Pr(\ell_i > y) = \lambda_j e^{-\lambda_j y} dy \prod_{i \neq j} e^{-\lambda_i y}.$$

Hence the joint density of (Y, J) is

$$f_{Y,J}(y, j) = \lambda_j e^{-\lambda_j y} \prod_{i \neq j} e^{-\lambda_i y} = \lambda_j e^{-\Lambda y}, \qquad y \geq 0,\ j \in \{1, \ldots, m\}.$$

The marginal density of Y by taking the limit as $dy \to 0$ is then

$$\begin{aligned}
f_Y(y) &= \sum_{i=1}^{m} f_{Y,J}(y, i) = \sum_{i=1}^{m} \lambda_i e^{-\Lambda y} = \Lambda e^{-\Lambda y} \\
\implies \Pr(J = j \mid Y = y) &= \frac{f_{Y,J}(y, j)}{f_Y(y)} = \frac{\lambda_j e^{-\Lambda y}}{\Lambda e^{-\Lambda y}} = \frac{\lambda_j}{\Lambda}.
\end{aligned}$$

Integrating the joint density over y gives the marginal of J:

$$\Pr(J = j) = \int_0^\infty f_{Y,J}(y, j)\, dy = \int_0^\infty \lambda_j e^{-\Lambda y}\, dy = \frac{\lambda_j}{\Lambda}.$$

Thus $f_{Y,J}(y, j) = f_Y(y) \Pr(J = j)$, so Y and J are independent. Finally, on the event $\{Y = y, J = j\}$, we must have $\ell_j = y$ and $\ell_i \geq y$ for all $i \neq j$ by definition of the minimum. □

Proof of Lemma 2: Memoryless decomposition of edge lengths

Lemma 2 (Memoryless decomposition of edge lengths)**.** *Conditional on the event $\{Y = y, J = j\}$, the minimum–attaining edge satisfies $\ell_j = y$, and every other edge length admits the decomposition*

$$\ell_i = y + A_i, \qquad i \neq j,$$

where A_i's denote the excess length beyond the minimum and each $A_i := \ell_i - y$ is independent of (Y, J) and is distributed as

$$A_i \sim \mathrm{Exp}(\lambda_i).$$

Moreover, the residuals $\{A_i : i \neq j\}$ are mutually independent.

Proof. On the event $\{Y = y, J = j\}$ we know $\ell_i \geq y$ for all $i \neq j$. For each such i,

$$A_i := \ell_i - y \;\big|\; (\ell_i \geq y)$$

has distribution $\mathrm{Exp}(\lambda_i)$ by the memoryless property of the exponential:

$$\Pr(A_i > t) = \Pr(\ell_i > y + t \mid \ell_i > y) = \frac{e^{-\lambda_i (y+t)}}{e^{-\lambda_i y}} = e^{-\lambda_i t}.$$

Independence of exponentials is preserved under truncation by the minimum event, so the A_i remain independent across $i \neq j$ and independent of (Y, J). □

We will also need the mean of the total "excess" length beyond the minimum.

Proof of Lemma 3: Mean excess length

Lemma 3 (Mean excess length). *Let A_i be as in Lemma 2, and let $X = \sum_{i\neq J} A_i$. Then*

$$\mathbb{E}[X] = \sum_{i=1}^{m} \frac{1}{\lambda_i} - \frac{m}{\Lambda}.$$

Proof. By Lemmas 1–2, for fixed j the random variables A_i for $i \neq j$ are independent with $A_i \sim \mathrm{Exp}(\lambda_i)$ and independent of J. Thus, $\mathbb{E}[X \mid J = j] = \mathbb{E}\left[\sum_{i\neq j} A_i \,\middle|\, J = j\right] = \sum_{i\neq j} \mathbb{E}[A_i] = \sum_{i\neq j} \frac{1}{\lambda_i}$. Using $\Pr(J = j) = \lambda_j/\Lambda$ (By Lemma 1), we obtain

$$\begin{aligned}
\mathbb{E}[X] &= \sum_{j=1}^{m} \Pr(J = j)\, \mathbb{E}[X \mid J = j] = \sum_{j=1}^{m} \frac{\lambda_j}{\Lambda} \sum_{i\neq j} \frac{1}{\lambda_i} \\
&= \sum_{j=1}^{m} \frac{\lambda_j}{\Lambda}\left(A - \frac{1}{\lambda_j}\right) = \frac{A}{\Lambda} \sum_{j=1}^{m} \lambda_j \; - \; \sum_{j=1}^{m} \frac{\lambda_j}{\Lambda} \cdot \frac{1}{\lambda_j} \\
&= A - \frac{m}{\Lambda} = \sum_{i=1}^{m} \frac{1}{\lambda_i} - \frac{m}{\Lambda}.
\end{aligned}$$

□

We now prove the conditional cover-time bound with respect to the minimum branch length using Lemmas 1–3.

Proof of Theorem 3: Conditional cover-time bound with respect to the minimum edge

Theorem 3 (Conditional cover-time bound with respect to the minimum edge). *For any $x > 0$,*

$$\mathbb{E}[T \mid Y \geq x] \;\leq\; H_m\left(m \;+\; \frac{1}{x}\Big(\sum_{i=1}^{m} \frac{1}{\lambda_i} - \frac{m}{\Lambda}\Big)\right).$$

Proof. We work throughout under the conditioning event $\{Y \geq x\}$ and explicitly mark this in expectations.

$$\begin{aligned}
\mathbb{E}[T \mid Y \geq x] &\leq H_m\, \mathbb{E}\left[\frac{1}{p_{\min}} \,\middle|\, Y \geq x\right] && \text{(nonuniform coupon collector with random probabilities)} \\
&= H_m\, \mathbb{E}\left[\frac{L_{\text{tot}}}{\ell_{\min}} \,\middle|\, Y \geq x\right] && (\text{since } p_{\min} = \ell_{\min}/L_{\text{tot}}) \\
&= H_m\, \mathbb{E}\left[\frac{\ell_{\min} + \sum_{i\neq J} \ell_i}{\ell_{\min}} \,\middle|\, Y \geq x\right] \\
&= H_m\, \mathbb{E}\left[1 + \frac{1}{\ell_{\min}} \sum_{i\neq J} (\ell_{\min} + A_i) \,\middle|\, Y \geq x\right] && (\text{Lemma 2: } \ell_i = \ell_{\min} + A_i \text{ for } i \neq J) \\
&= H_m\, \mathbb{E}\left[m + \frac{1}{\ell_{\min}} \sum_{i\neq J} A_i \,\middle|\, Y \geq x\right] \\
&= H_m\left(m + \mathbb{E}\left[\frac{1}{\ell_{\min}} \,\middle|\, Y \geq x\right] \cdot \mathbb{E}\left[\sum_{i\neq J} A_i\right]\right) && \Big(\textstyle\sum_{i\neq J} A_i \text{ is independent of } Y \text{ and } J\Big) \\
&= H_m\left(m + \mathbb{E}\left[\frac{1}{\ell_{\min}} \,\middle|\, Y \geq x\right]\left(\sum_{i=1}^{m} \frac{1}{\lambda_i} - \frac{m}{\Lambda}\right)\right) && \text{(by Lemma 3)} \\
&\leq H_m\left(m + \frac{1}{x}\left(\sum_{i=1}^{m} \frac{1}{\lambda_i} - \frac{m}{\Lambda}\right)\right) && (\text{on } \{Y \geq x\} : 1/\ell_{\min} \leq 1/x \text{ a.s.})
\end{aligned}$$

□

Proof of Corollary 1: High-probability cover-time bound

Corollary 1 (High-probability cover-time bound). *Let $\delta \in (0,1)$ be a desired failure probability when $Y < x$ and so, $\Pr(Y \geq x) = 1 - \delta$. Following this,*

$$x = \frac{1}{\Lambda} \log \frac{1}{1-\delta}.$$

and on this $(1-\delta)$-probability event,

$$\mathbb{E}[T \mid Y \geq x] \;\leq\; H_m\left(m + \frac{\Lambda}{\log(1/(1-\delta))}\Big(\sum_{i=1}^{m} \frac{1}{\lambda_i} - \frac{m}{\Lambda}\Big)\right).$$

Proof. We interpret δ as the failure probability $\delta = \Pr(Y < x)$. Since $Y \sim \text{Exp}(\Lambda)$ by Lemma 1, we have

$$\Pr(Y \geq x) = 1 - \Pr(Y < x) = 1 - \delta = e^{-\Lambda x}.$$

Solving $1 - \delta = e^{-\Lambda x}$ for x gives $x = \frac{1}{\Lambda} \log \frac{1}{1-\delta}$. Substituting x in Theorem 3 yields

$$\mathbb{E}[T \mid Y \geq x] \;\leq\; H_m \left(m + \frac{\Lambda}{\log(1/(1-\delta))} \Big(\sum_{i=1}^{m} \frac{1}{\lambda_i} - \frac{m}{\Lambda} \Big) \right)$$

□

Proof of Theorem 4: Expected cover time under a simplified 1-Dollo model

Theorem 4 (Expected cover time under a simplified 1-Dollo model)**.** *Under the 1-Dollo assumption, each mutation is gained once and may be lost at most once on a descendant branch of the gain. A single mutation can therefore cover either one previously uncovered edge (gain-only) or two uncovered edges (gain–loss). Although the tree-constrained 1-Dollo model requires the loss edge to be descendant from the gain edge, for analytical tractability we derive the following bound under a simplified independent coverage model, in which the gain and loss edges are treated as independently selected edges. Let $p = P_{\text{loss}} \in [0, 1)$ denote the probability that a gain undergoes loss. Then the expected number of mutations required to cover all m edges is*

$$\mathbb{E}[T] = \frac{m}{1+p} \left(H_m - \sum_{k=1}^{m} \frac{(-p)^{m-k+1}}{k} \right), \qquad H_m = \sum_{k=1}^{m} \frac{1}{k} \tag{6}$$

Proof. Let's consider the process of sequentially assigning mutations to the branches of $\mathcal{T}$. Let i denote the number of edges that are already covered by at least one mutation (equivalently, the number of mutations mapped so far). When a new mutation is sampled, three outcomes are possible: it hits (1) an uncovered gain-only edge, covering one new edge; (2) an uncovered gain–loss pair, covering two new edges; or (3) an already covered edge, covering none. The corresponding state transitions are shown in Figure S1.

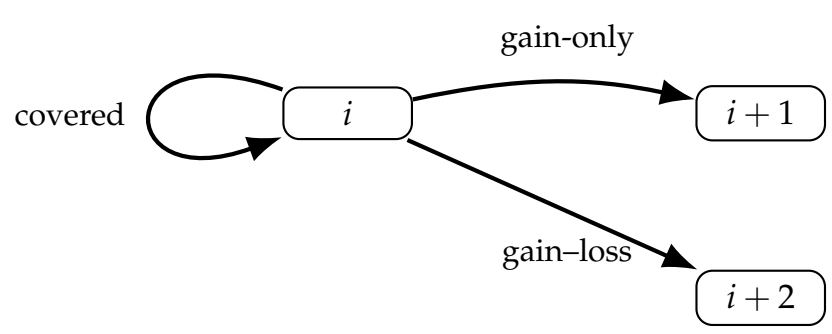

Fig. S1. State transitions under the 1-Dollo model. Each new mutation may cover one (gain-only) or two (gain–loss) new edges, or none (already covered).

Let $u_i = \frac{m-i}{m}$ be the probability that the sampled mutation lands on an uncovered edge. Then, $u_i p$ will be the probability that the mapped mutation will also have a loss associated with it. Let $P_1 = u_i(1-p), P_2 = u_i p, P_0 = 1 - u_i$, corresponding respectively to transitions $i \to i+1$, $i \to i+2$, and $i \to i$. Let $E_i = \mathbb{E}[T_i]$ denote the expected number of remaining mutations given i covered edges. This follows the recurrence

$$\begin{aligned} E_i &= 1 + P_1 E_{i+1} + P_2 E_{i+2} + P_0 E_i \\ \implies E_i &= \frac{1 + P_1 E_{i+1} + P_2 E_{i+2}}{P_1 + P_2} \\ &= \frac{1}{u_i} + (1-p) E_{i+1} + p E_{i+2} \end{aligned}$$

where, $E_m = 0$ is the base case. Let $r = m - i$ be the number of uncovered edges and define $A_r = E_{m-r}$, where A_r is a reparameterization of E_{m-r} denoting the expected number of mutations when r edges are remaining to be mapped. Then, $u_i = r/m$ and $A_r = \frac{m}{r} + (1-p)A_{r-1} + pA_{r-2}$ where the base cases are $A_0 = 0,\ A_1 = m$. Defining $S_r = A_r - A_{r-1}$ gives $S_r - pS_{r-1} = \frac{m}{r}$ with base case, $S_1 = m$. Solving the recurrence, we get:

$$\begin{aligned} S_r &= m(-p)^{r-1} + m \sum_{k=2}^{r} \frac{(-p)^{r-k}}{k} \\ \implies A_r &= m \sum_{j=1}^{r} (-p)^{j-1} + m \sum_{j=1}^{r} \sum_{k=2}^{j} \frac{(-p)^{j-k}}{k} && \Big(\text{Since } A_r = \sum_{j=1}^{r} S_j\Big) \end{aligned}$$

The first term is geometric, so $\sum_{j=1}^{r} (-p)^{j-1} = \frac{1-(-p)^r}{1+p}$. For the double sum,

$$\sum_{j=1}^{r} \sum_{k=2}^{j} \frac{(-p)^{j-k}}{k} = \sum_{k=2}^{r} \frac{1}{k} \sum_{j=k}^{r} (-p)^{j-k} = \sum_{k=2}^{r} \frac{1}{k} \cdot \frac{1-(-p)^{r-k+1}}{1+p}.$$

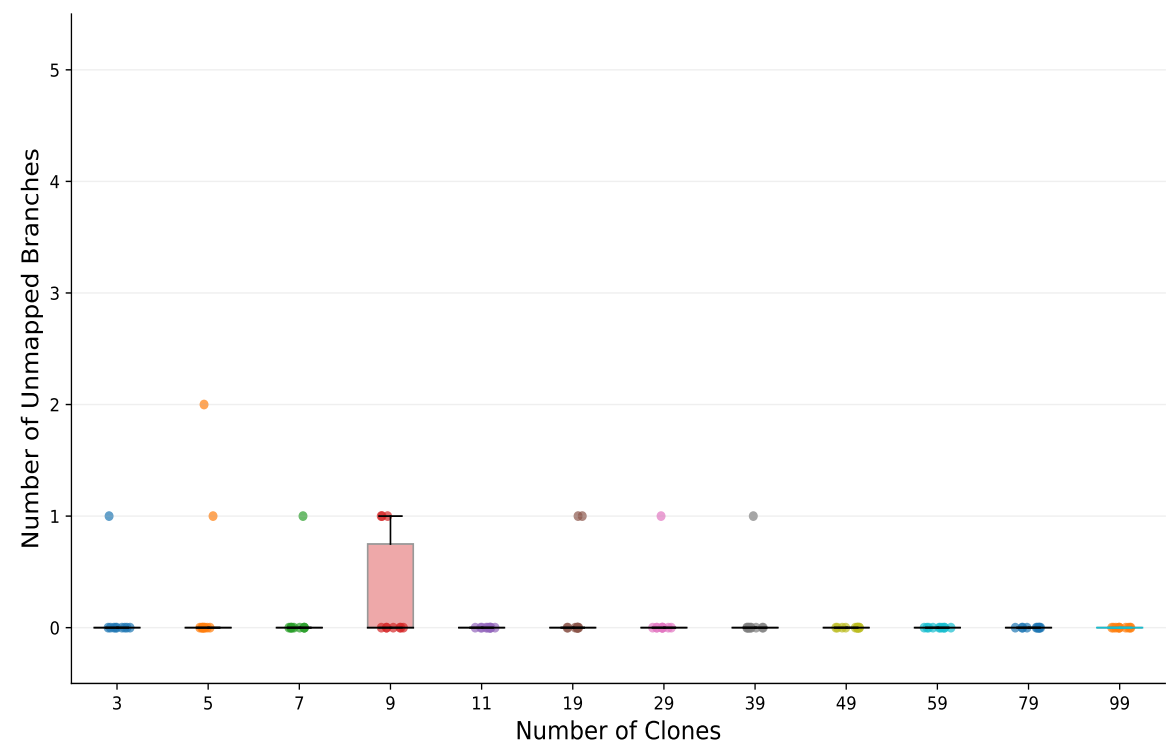

Fig. S2. Results on the number of unmapped branches for perfect phylogenies with $\{3, 5, 7, 9, 11, 19, 29, 39, 49, 59, 79, 99\}$ clones.

Hence

$$\begin{aligned} A_r &= \frac{m}{1+p}\left[\left(1-(-p)^r\right)+\sum_{k=2}^{r}\frac{1}{k}-\sum_{k=2}^{r}\frac{(-p)^{r-k+1}}{k}\right] \\ &= \frac{m}{1+p}\left[\left(1+\sum_{k=2}^{r}\frac{1}{k}\right)-\left((-p)^r-\sum_{k=2}^{r}\frac{(-p)^{r-k+1}}{k}\right)\right] \\ &= \frac{m}{1+p}\left(H_r-\sum_{k=1}^{r}\frac{(-p)^{r-k+1}}{k}\right) \qquad (\text{Since } H_r=\sum_{k=1}^{r}\frac{1}{k}) \end{aligned}$$

Starting from no covered edges ($i = 0, r = m$),

$$\mathbb{E}[T] = A_m = \frac{m}{1+p}\left(H_m-\sum_{k=1}^{m}\frac{(-p)^{m-k+1}}{k}\right) \qquad \square$$

Supplementary Results

Evaluation Criteria

Pairwise Ancestral Accuracy.

Given the ground-truth tree $\mathcal{T}$ and an inferred tree $\mathcal{T}'$, we consider every pair of mutations (a, b) that appear in both trees. For each tree, we determine whether mutation a is ancestral to b, b is ancestral to a, or if the two mutations are independent (i.e., neither is ancestral to the other). The pairwise ancestral relationship accuracy is then defined as the fraction of mutation pairs for which the inferred tree $\mathcal{T}'$ assigns the same ancestral relationship as the ground-truth tree $\mathcal{T}$. Formally,

$$\text{Accuracy} = \frac{|\{(a,b) : r_{\mathcal{T}}(a,b) = r_{\mathcal{T}'}(a,b)\}|}{\binom{M}{2}},$$

where M is the number of mutations shared between $\mathcal{T}$ and $\mathcal{T}'$, and corresponds to the subset of mutations from $\mathcal{T}$ after subsampling. $r_{\mathcal{T}}(a, b) \in \{\text{ancestor, descendant, independent}\}$ denotes the ancestral relationship between a and b in tree $\mathcal{T}$.

Perfect and 1-Dollo simulations

To evaluate the robustness of our bounds for perfect and 1-Dollo simulations, we first generated perfect phylogenies with $n \in \{2, 3, 4, 5, 6, 10, 15, 20, 25, 30, 40, 50\}$ leaves. This corresponds to $(2n - 1) \in \{3, 5, 7, 9, 11, 19, 29, 39, 49, 59, 79, 99\}$ total nodes or clones in the tree. Since a full binary tree with n leaves has $m = 2n - 2$ edges, we generated mH_m mutations and mapped each of these mH_m mutations to a uniformly chosen edge in the tree. After assigning all mutations, we counted how many branches of the perfect phylogeny remained without any mutation. We simulated 10 replicates for each value of n. Figure S2 shows the distribution of the number of unmapped branches. The results indicate that using the perfect-phylogeny bound almost always results in every branch receiving at least one mutation.

K-dollo phylogeny reconstruction accuracies for different loss probabilities

We simulated k-Dollo phylogenies with loss probabilities $p \in \{0.2, 0.3, 0.4\}$, following the procedure described in Section 3.1. Simulations were conducted for clone counts $\{3, 5, 7, 9, 11, 19, 29\}$, with the number of sampled mutations set as a multiple $x \in \{0.05, 0.1, 0.5, 0.7, 1, 2, 3\}$ of the perfect-phylogeny bound. For each combination of parameters, we generated 10 independent instances and used SPhyR El-Kebir [2018] to reconstruct the corresponding k-Dollo phylogenies. Figures S3, S4, and S5 correspond to loss probabilities $p = 0.2$, $p = 0.3$, and $p = 0.4$, respectively, and show trends consistent with the $p = 0.1$ case described in the main text. However, as both the loss probability and k increase, SPhyR El-Kebir [2018] requires a larger number of sampled mutations to accurately reconstruct the phylogenies.

Figure S6 shows the fraction of true branches covered by the sampled mutations across different clone counts, for both the perfect phylogeny case and the 1-Dollo case with loss probabilities $p \in \{0.1, 0.2, 0.3, 0.4\}$.

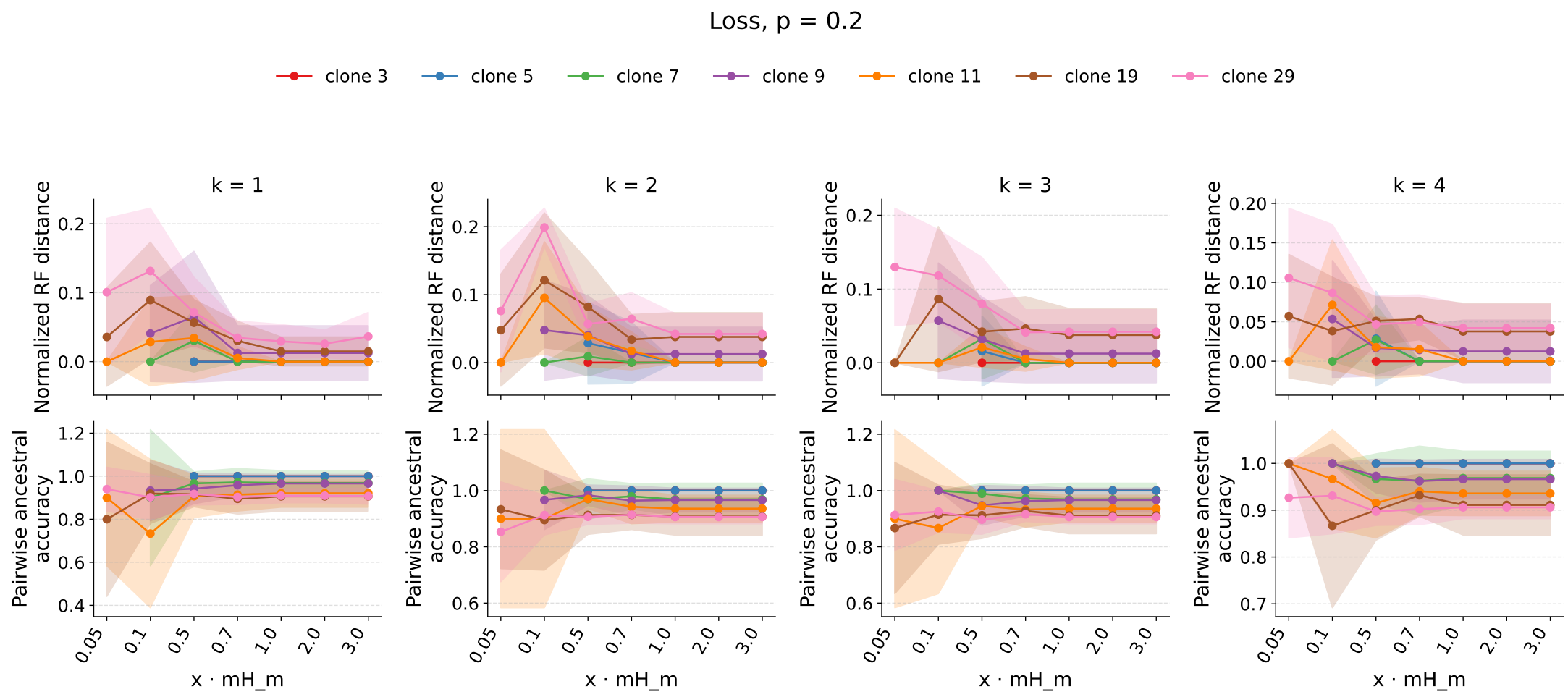

Fig. S3. Results for k-Dollo phylogeny reconstruction with SPhyR. The figure shows normalized phylogeny inference (top) and pairwise ancestral accuracy (bottom) across a range of k, clone numbers, and sampling rates for loss probability, $p = 0.2$.

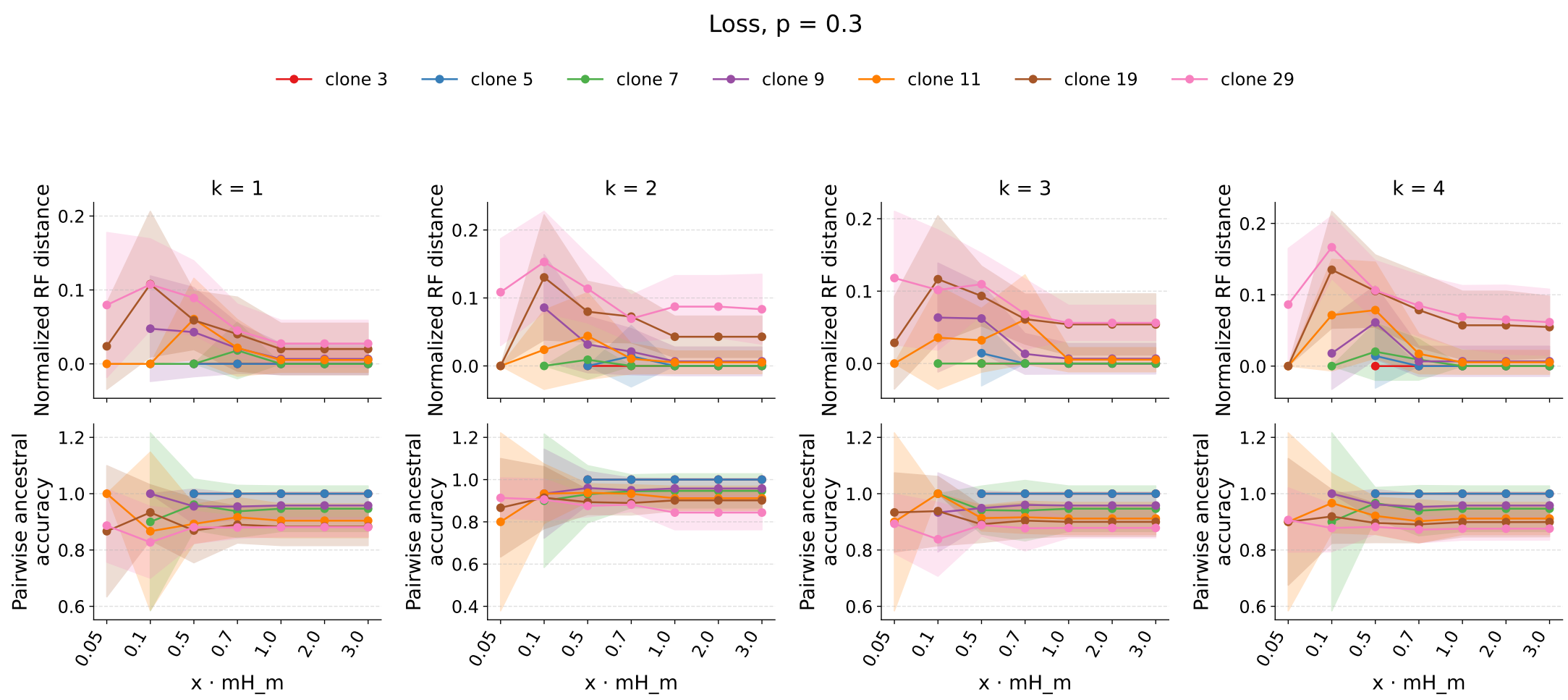

Fig. S4. Results for k-Dollo phylogeny reconstruction with SPhyR. The figure shows normalized phylogeny inference (top) and pairwise ancestral accuracy (bottom) across a range of k, clone numbers, and sampling rates for loss probability, $p = 0.3$.

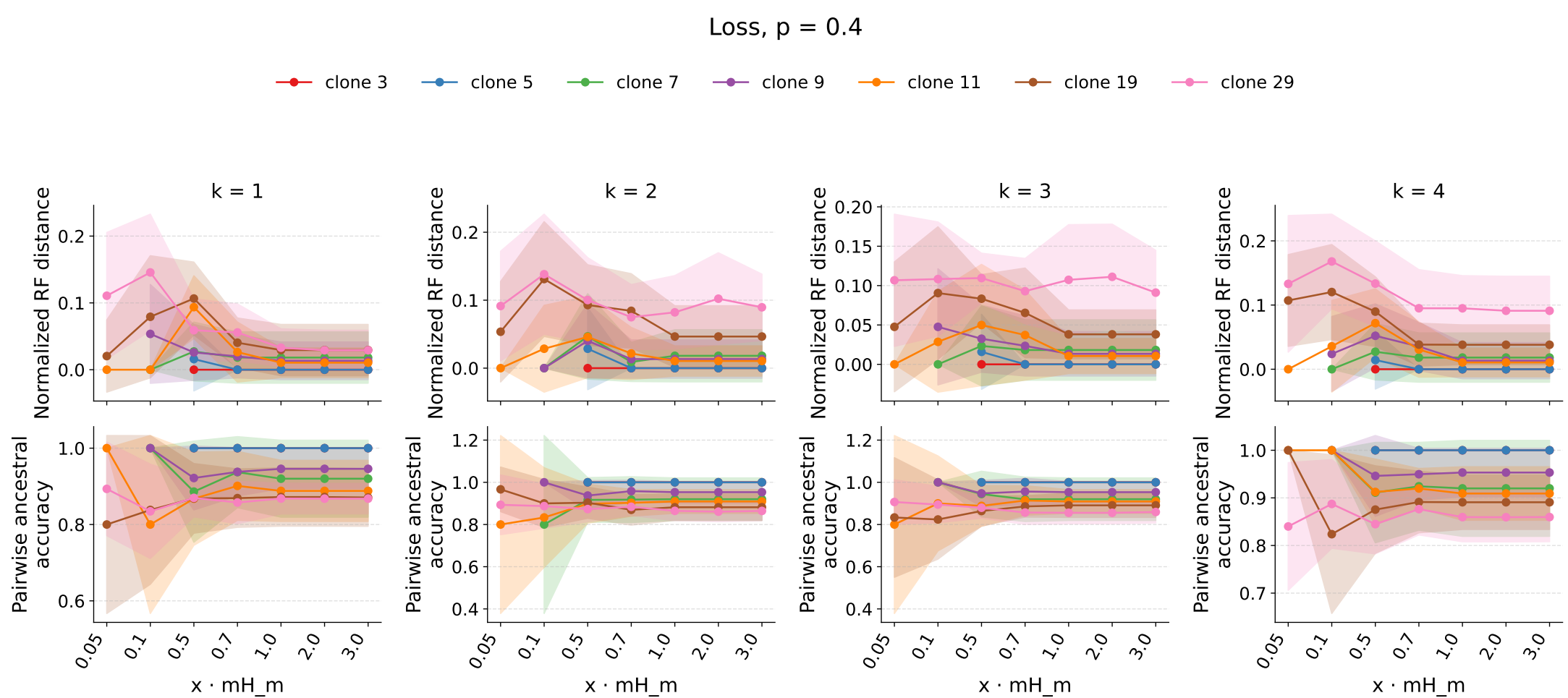

Fig. S5. Results for k-Dollo phylogeny reconstruction with SPhyR. The figure shows normalized phylogeny inference (top) and pairwise ancestral accuracy (bottom) across a range of k, clone numbers, and sampling rates for loss probability, $p = 0.4$.

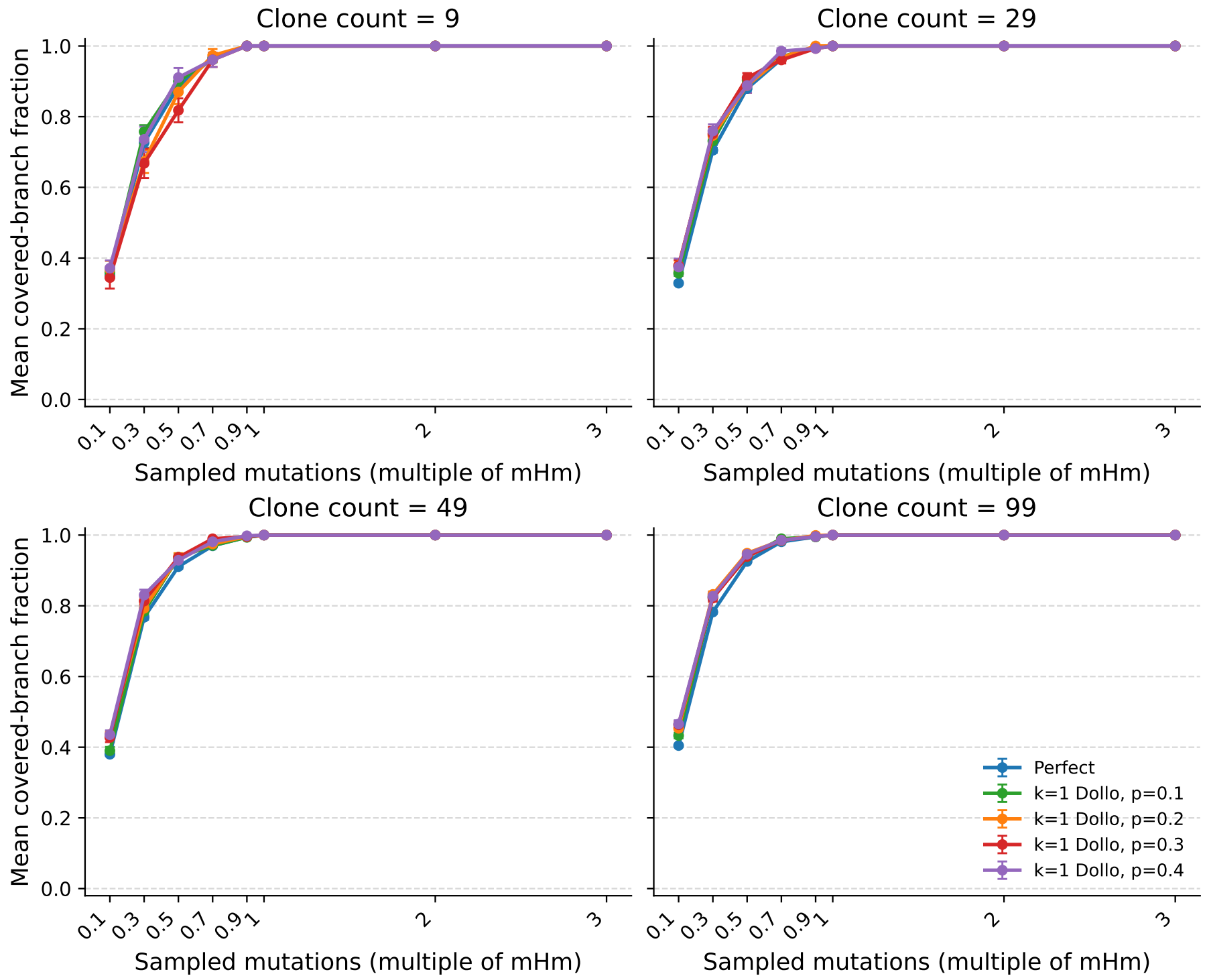

Fig. S6. Mean fraction of true branches covered by the sampled mutations for $n \in \{9, 29, 49, 99\}$, plotted against the number of sampled mutations as a multiple of mH_m. The curves correspond to the perfect phylogeny case and k=1 Dollo trees with $p \in \{0.1, 0.2, 0.3, 0.4\}$.

Robustness of the bounds under noisy settings

To test the robustness of our sampling bounds under noisy settings to represent real data, we performed additional simulations with fixed false-positive (FP) and false-negative (FN) rates, and show the results in Figures S7-S11. Specifically, we simulated 1-Dollo phylogenies with the number of clones in $\{3, 5, 7, 9, 11\}$ and considered three noise settings, $(fp, fn) \in \{(0.5\%, 0.5\%), (1\%, 1\%), (2\%, 2\%), (5\%, 5\%)\}$. For each setting, we sampled x mHm mutations, where $x \in \{0.5, 0.7, 1, 2, 3\}$ with mutation loss probability $p = 0.1$.

As expected, reconstruction error generally increased with both the number of clones and the error rate, reflecting the greater difficulty of resolving more complex phylogenies from noisy observations. For trees with three or five clones, the mean normalized Robinson–Foulds distance remained below 0.1 across most of the tested settings. More generally, the reconstructed trees remained largely accurate for combined FP and FN rates of up to 4%, with mean normalized Robinson-Foulds distances generally at or below 0.2.

At a fixed error rate, increasing the number of sampled mutations also increases the absolute number of erroneous observations. Thus, although additional mutations provide more phylogenetic signal, they may also introduce more false-positive and false-negative entries into the reconstruction problem. Nevertheless, the overall reconstruction accuracy remained robust under moderate noise.

At the highest tested noise level, corresponding to a combined FP and FN rate of 10%, reconstruction became substantially more difficult for SPhyR. In several simulations, SPhyR was unable to resolve a tree. This was particularly pronounced for the 11-clone setting, for which no consistent tree could be found. For trees with 3, 5, 7, or 9 clones, however, the results followed the same general pattern observed at lower noise levels, although reconstruction errors were higher than lower total error rate simulations. Overall, these results suggest that the mutation-sampling scales predicted by our theoretical bounds remain practically informative and support robust phylogenetic reconstruction in the presence of moderate observational noise.

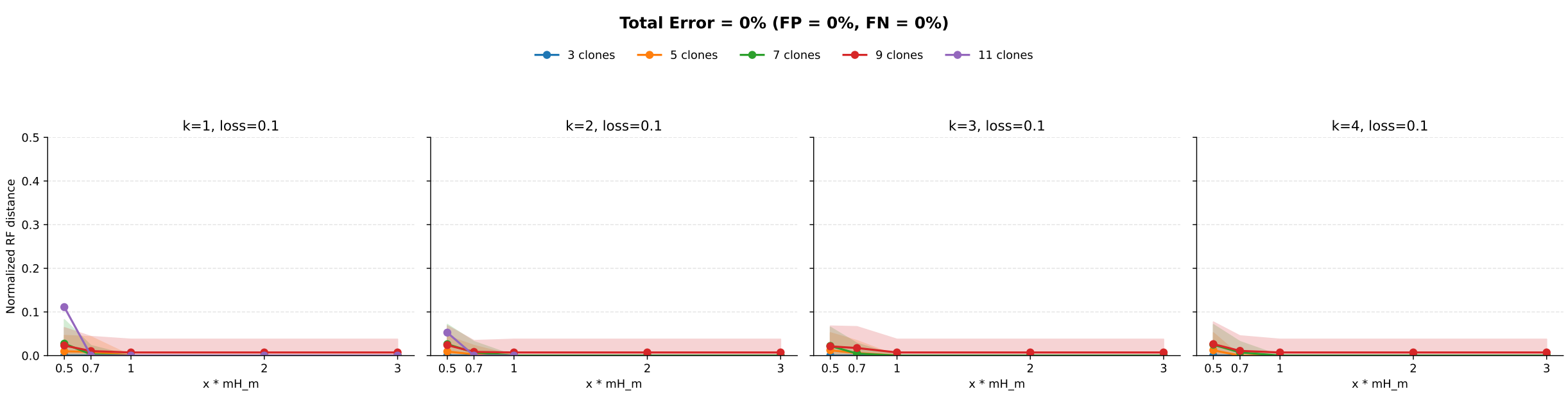

Fig. S7. Results for 1-Dollo phylogeny reconstruction using SPhyR with mutation-loss probability $p = 0.1$ and a 0% sequencing error rate. The figure shows the normalized Robinson-Foulds distance for different mutation-sampling levels $x \cdot mH_m$, where $x \in \{0.5, 0.7, 1, 2, 3\}$, across phylogenies containing 3, 5, 7, 9, and 11 clones.

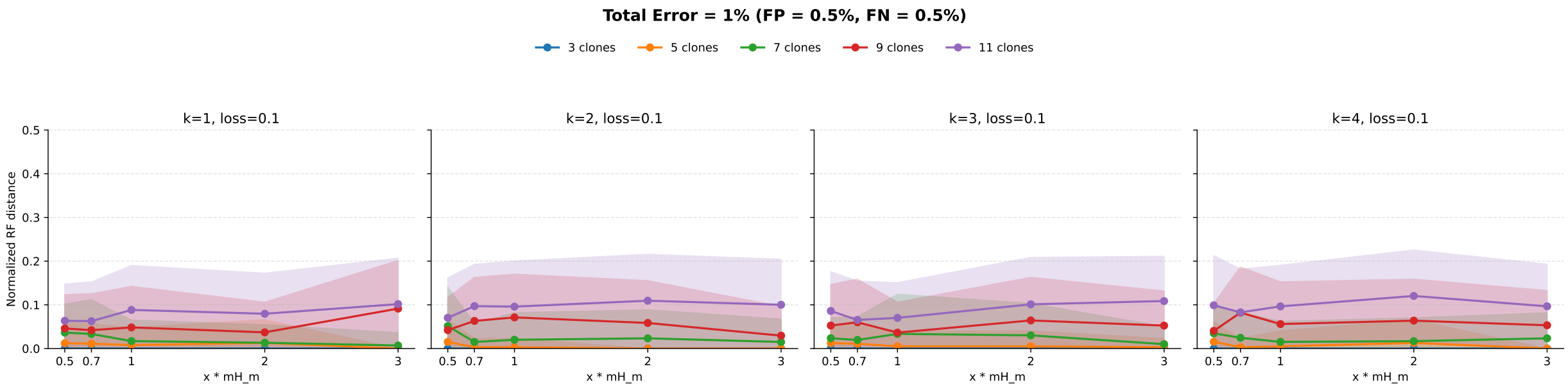

Fig. S8. Results for 1-Dollo phylogeny reconstruction using SPhyR with mutation-loss probability $p = 0.1$ and a 1% sequencing error rate. The figure shows the normalized Robinson-Foulds distance for different mutation-sampling levels $x \cdot mH_m$, where $x \in \{0.5, 0.7, 1, 2, 3\}$, across phylogenies containing 3, 5, 7, 9, and 11 clones.

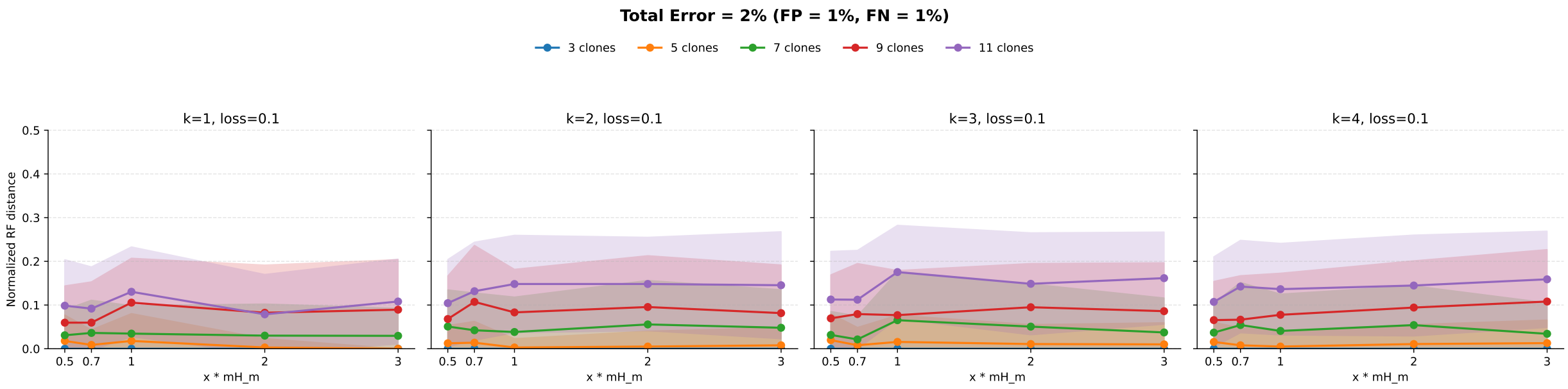

Fig. S9. Results for 1-Dollo phylogeny reconstruction using SPhyR with mutation-loss probability $p = 0.1$ and a 2% sequencing error rate. The figure shows the normalized Robinson-Foulds distance for different mutation-sampling levels $x \cdot mH_m$, where $x \in \{0.5, 0.7, 1, 2, 3\}$, across phylogenies containing 3, 5, 7, 9, and 11 clones.

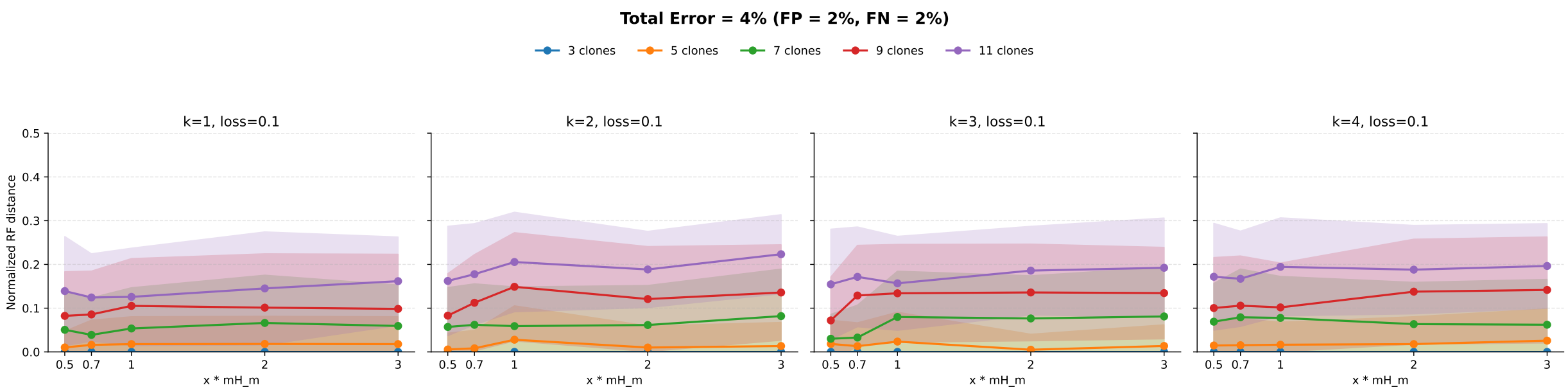

Fig. S10. Results for 1-Dollo phylogeny reconstruction using SPhyR with mutation-loss probability $p = 0.1$ and a 4% sequencing error rate. The figure shows the normalized Robinson-Foulds distance for different mutation-sampling levels $x \cdot mH_m$, where $x \in \{0.5, 0.7, 1, 2, 3\}$, across phylogenies containing 3, 5, 7, 9, and 11 clones.

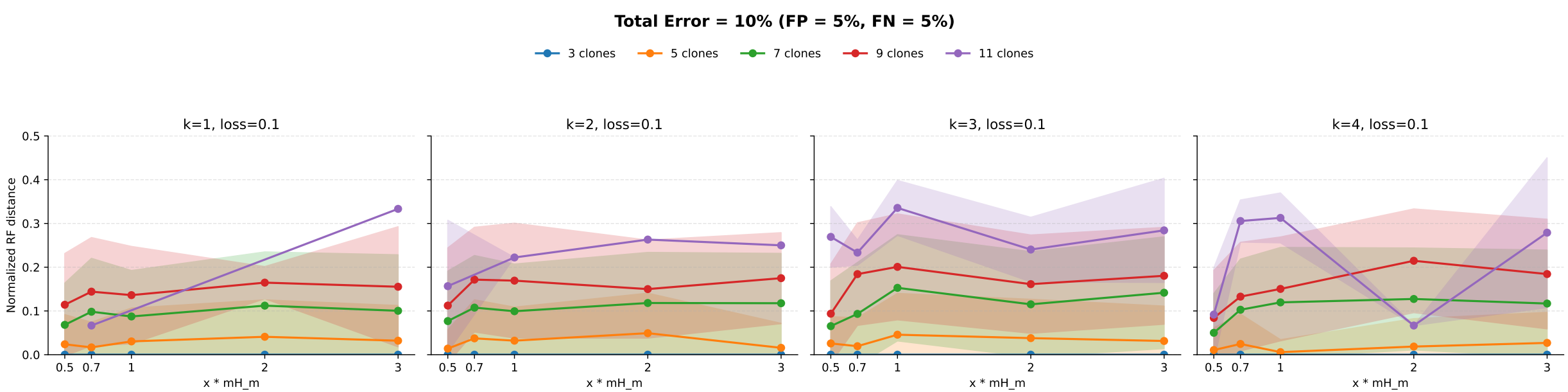

Fig. S11. Results for 1-Dollo phylogeny reconstruction using SPhyR with mutation-loss probability $p = 0.1$ and a 10% sequencing error rate. The figure shows the normalized Robinson-Foulds distance for different mutation-sampling levels $x \cdot mH_m$, where $x \in \{0.5, 0.7, 1, 2, 3\}$, across phylogenies containing 3, 5, 7, 9, and 11 clones.

Expected number of mutations and high probability bounds for different number of edges in the perfect phylogeny setting

Here, we show show how the expected number of mutations and the corresponding high-probability bound vary with the number of edges in the perfect phylogeny setting. Figure S12 (a) shows mH_m, which gives the expected number of mutations sufficient to cover all edges, for representative values $n \in \{3, 4, 5, 8, 10, 15, 20, 30\}$. mH_m increases with m, showing the increase in expected number of mutations as the number of edges in the tree increases.

Figure S12 (b) shows the high-probability bound $\Pr(T > t) \leq me^{-t/m}$, plotted as a function of the multiple c in $t = c * mH_m$. This shows how the tail probability decreases as the number of sampled mutations increases beyond the baseline scale mH_m. The dashed vertical line at $c = 1$ is for $t = mH_m$, while larger values of c correspond to increasingly stronger coverage guarantees. We see that, across the values of m considered, the tail probability becomes small beginning around $c = 2$, indicating that sampling at roughly twice the expectation-level bound provides a substantially stronger high-probability guarantee.

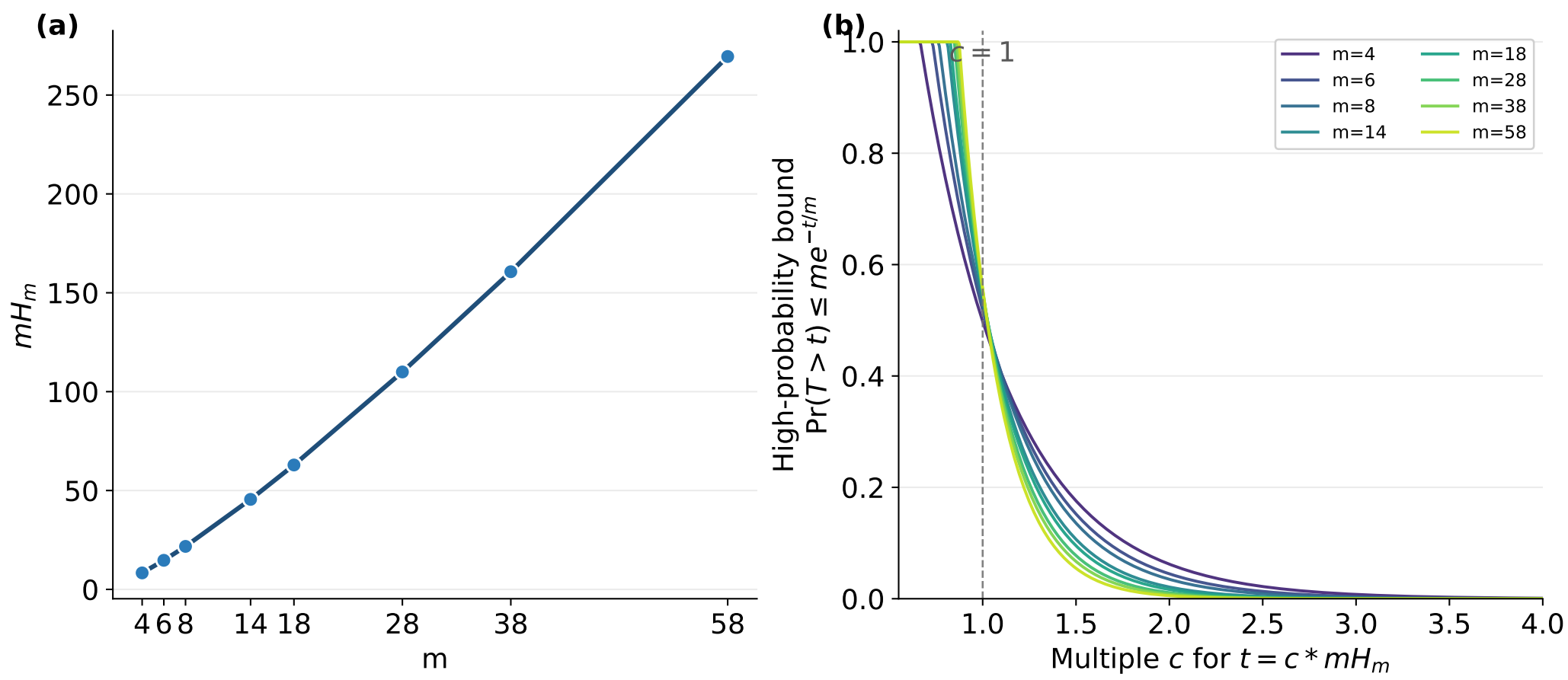

Fig. S12. (a) The quantity mH_m as a function of m, where $m = 2n - 2$ for $n \in \{3, 4, 5, 8, 10, 15, 20, 30\}$. (b) The upper bound $p(T > t) \leq me^{-t/m}$ plotted against the multiple c in $t = c * mH_m$ for the same set of m values. The dashed vertical line shows $c = 1$, corresponding to $t = mH_m$.

Summary of the theoretical bounds and the expectation each result targets

Supplementary Table S1 summarizes the sufficient numbers of sampled mutations predicted by our theoretical results for the perfect-phylogeny and 1-Dollo models. We report these values for a range of numbers of leaves n, the corresponding numbers of edges m, and for perfect phylogeny, and for 1-Dollo loss probabilities $p \in \{0.1, 0.2, 0.3, 0.4\}$. The table is intended to provide a practical reference for choosing mutation sampling levels under the evolutionary models analyzed in this work.

n	m	Perfect	$p = 0.1$	$p = 0.2$	$p = 0.3$	$p = 0.4$
2	2	3	3	3	3	3
3	4	9	8	8	7	7
4	6	15	14	13	12	11
5	8	22	20	19	17	16
6	10	30	27	25	23	22
10	18	63	58	53	49	46
15	28	110	101	92	85	79
20	38	161	147	135	124	115
25	48	215	195	179	165	154
30	58	270	246	225	208	193
40	78	386	351	322	297	276
50	98	507	461	423	390	362
100	198	1162	1057	969	894	831

Table S1. Expected number of sampled mutations, $\mathbb{E}[T]$, for perfect phylogeny and 1-Dollo models with different loss probabilities $p \in \{0.1, 0.2, 0.3, 0.4\}$. Here, n denotes the number of leaves and m denotes the number of edges.

Runtime analysis

In this section, we examine how the runtime of SPhyR varies across simulation settings with different numbers of clones by comparing inference on the full mutation sets with inference on the corresponding sampled mutation sets. Although SPhyR converged relatively quickly on these simulation instances (Figure S13), we consistently observed that the full datasets required more runtime than the sampled datasets.

We further extended the runtime analysis of SphyR to Sc-TUSV-ext [Bristy et al., 2025], which has runtime scaling exponentially with the number of sampled mutations. Sc-TUSV-ext reconstructs tumor evolutionary histories from SNVs, CNVs, and SVs, and therefore requires a more complex

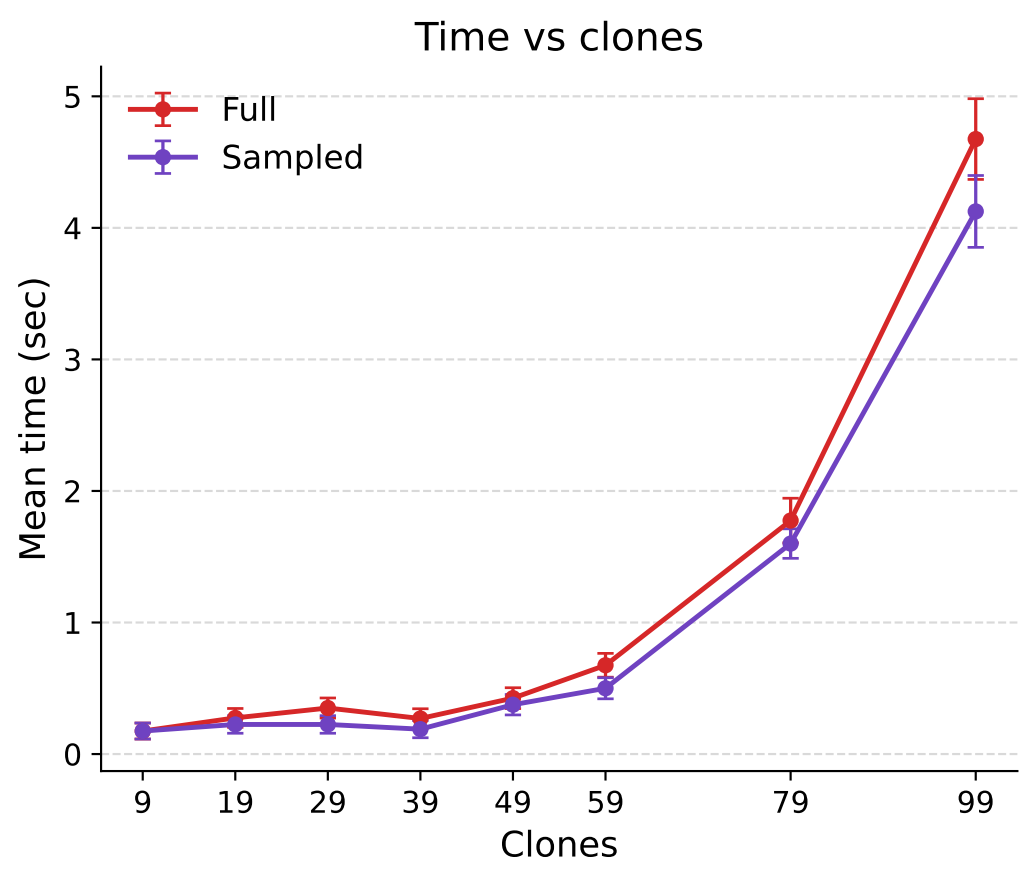

Fig. S13. Runtime analysis for reconstructing full (red) vs sampled (blue) set of mutation phylogenies with SphyR. We ran SphyR with total number of clones in the tree, $(2n-1) \in \{9, 19, 29, 39, 49, 59, 79, 99\}$ for $n \in \{5, 10, 15, 20, 25, 30, 40, 50\}$

.

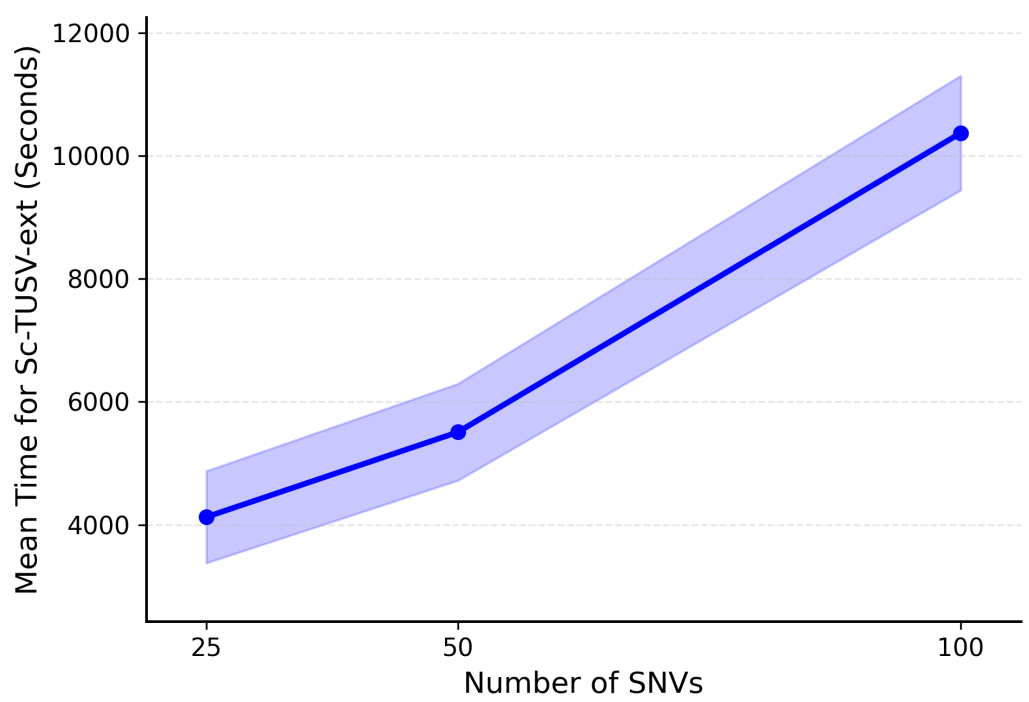

Fig. S14. Runtime analysis of evolutionary tree reconstruction with Sc-TUSV-ext as the number of mutations varies. We simulated 10 instances for each setting with 25, 50, and 100 SNVs. The x-axis shows the number of SNVs, and the y-axis shows the runtime of Sc-TUSV-ext in seconds.

evolutionary model for which algorithms scale more poorly in the number of variants, which the intended use case for the present method. Figure S14 shows how the runtime of Sc-TUSV-ext changes as the number of sampled mutations increases. We simulated 10 Dollo phylogeny instances with a 5-clone tree and 25, 50, or 100 subsampled SNV mutations. We observe that the runtime increases with the number of subsampled mutations.

Additional evaluation on a high grade serous ovarian cancer patient

To further validate the theoretical bounds on a real dataset analysis beyond CRC1 in the main paper, we analyzed an additional high-grade serous ovarian cancer patient sample, DG1134, from Funnell et al. [2022]. The data set contained 113 single cells and 15,642 SNVs. After filtering out SNVs with total read count < 4, 3,459 SNVs remained for the downstream analysis. We first clustered the cells into 4, 5, 6, and 7 clones with K-means clustering. The 5, 6, and 7 clone solutions each contained at least one singleton clone, whereas the 4 clone solution yielded clone sizes of 23, 36, 50, and 4 cells. We therefore focused the downstream analysis on the 4 clone case and randomly selected one cell from each clone as a clone representative.

For the 4-clone solution, corresponding to a tree with $n = 4$ leaves and $m = 2n - 2 = 6$ edges, the equal-branch-length perfect-phylogeny bound gives $mH_m = 15$ sampled SNVs. To assess robustness around this bound, we also considered a more conservative sampling level of $2mH_m = 30$ sampled SNVs. For each sampling level, we generated 100 replicates and reconstructed trees using both PAUP and SPhyR, comparing the trees inferred from sampled mutations with those inferred from the full mutation set.

We additionally interpret these results in the context of the high-probability bound introduced in the revised manuscript. Under the equal-branch-length perfect-phylogeny model,

$$\Pr(T > t) \leq me^{-t/m}.$$

For $t = 15$, this gives $\Pr(T > 15) \leq 6e^{-15/6} \approx 0.49$, indicating that the expectation-level bound is loose and it alone does not guarantee highly reliable mutation coverage. For $t = 30$, the bound improves to $\Pr(T > 30) \leq 6e^{-30/6} \approx 0.04$. We therefore report results for both $t = 15$ and $t = 30$ in order to evaluate performance both at the theoretical expectation and at a more conservative, high-probability sampling level.

We show these results in Figure S15. PAUP showed occasional mismatches between the sampled-mutation trees and the full-mutation tree when t=15, whereas for t=30 it consistently recovered the same topology as that obtained from the full mutation set. We ran SPhyR under the 1-Dollo setting to account for the possibility of mutation losses in the real patient data, and observed that the branches in the full-mutation tree were supported by highly variable numbers of mutations. Since our theoretical analysis for the perfect-phylogeny and 1-Dollo settings assumes a relatively uniform distribution of mutations across branches, we additionally used a mutation-weighted RF distance as a comparison metric, in which the contribution

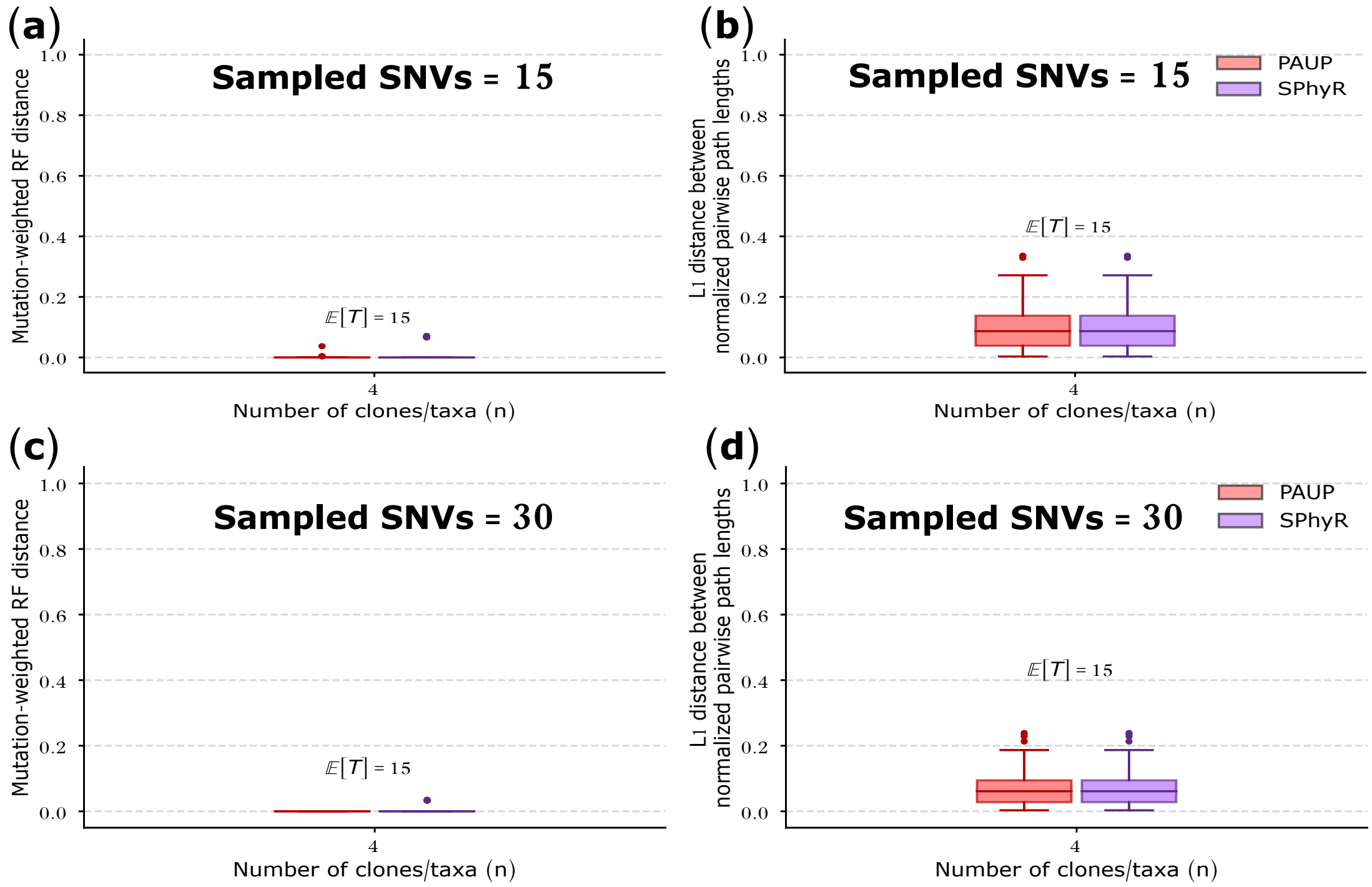

Fig. S15. Results for patient DG1134 with n = 4. For each setting, we generated 100 replicates, reconstructed the full mutation trees using PAUP and SPhyR, and compared them with trees inferred from subsampled mutations. (a,c) Weighted Robinson–Foulds (RF) distance between the full-mutation and sampled-mutation trees for 15 and 30 sampled SNVs, respectively. (b,d) L1 distance between the normalized pairwise path lengths of the full-mutation and sampled-mutation trees for 15 and 30 sampled SNVs, respectively.

of each branch was weighted by the fraction of all tree mutations mapped to that branch. Under this adjusted comparison, the trees reconstructed by SPhyR from sampled mutations agreed with the full-data tree in most replicates for both t=15 and t=30, with the t=30 case showing higher accuracy. The normalized pairwise path-length L1 distances remained small and decreased at the higher sampling level (Figure S15b,d). Overall, these additional results provide a stronger real-data validation than the CRC1 analysis and suggest that the theoretical bound is informative in practice, while a modest multiple of the bound yields more stable reconstruction.
